# SARS-CoV-2 seroepidemiology in Mongolia, 2020–2021: a longitudinal national study

**DOI:** 10.1016/j.lanwpc.2023.100760

**Published:** 2023-04-10

**Authors:** Battogtokh Chimeddorj, Christopher R. Bailie, Undram Mandakh, David J. Price, Batzorig Bayartsogt, Niamh Meagher, Oyunbaatar Altanbayar, Battur Magvan, Zolzaya Deleg, Anuujin Gantumur, Otgonjargal Byambaa, Enkhgerel Nyamdavaa, Khangai Enkhtugs, Usukhbayar Munkhbayar, Batkhuu Bayanjargal, Tuyajargal Badamsambuu, Myagmartseren Dashtseren, Tsolmontuya Amartuvshin, Zolmunkh Narmandakh, Khongorzul Togoo, Enkh-Amar Boldbaatar, Ariunzaya Bat-Erdene, Usukhbayar Chimeddorj, Khurelbaatar Nyamdavaa, Erdembileg Tsevegmid, Ochbadrakh Batjargal, Oyunsuren Enebish, Gerelmaa Enebish, Batzaya Batchuluun, Gereltsetseg Zulmunkh, Ganbaatar Byambatsogt, Temuulen Enebish, Linh-Vi Le, Isabel Bergeri, Jodie McVernon, Ryenchindorj Erkhembayar

**Affiliations:** a Department of Microbiology and Infection Prevention Control, School of Biomedicine, Mongolian National University of Medical Sciences, Ulaanbaatar, Mongolia; b Institute of Biomedical Sciences, Mongolian National University of Medical Sciences, Ulaanbaatar, Mongolia; c Department of Infectious Diseases, Peter Doherty Institute for Infection and Immunity, The University of Melbourne, Melbourne, Australia; d Department of Family Medicine, School of Medicine, Mongolian National University of Medical Sciences, Ulaanbaatar, Mongolia; e Centre for Epidemiology & Biostatistics, Melbourne School of Population & Global Health, The University of Melbourne, Melbourne, Australia; f Department of Epidemiology and Biostatistics, School of Public Health, Mongolian National University of Medical Sciences, Ulaanbaatar, Mongolia; g Department of Immunology, School of Biomedicine, Mongolian National University of Medical Sciences, Ulaanbaatar, Mongolia; h Mongolian National University of Medical Sciences, Ulaanbaatar, Mongolia; i Ministry of Health, Mongolia; j Department of Molecular Biology and Genetics, School of Biomedicine, Mongolian National University of Medical Sciences, Ulaanbaatar, Mongolia; k Department of Research Planning, Ministry of Health, Mongolia; l Central Clinical Laboratory, Mongolia Japan Hospital, Mongolian National University of Medical Sciences, Ulaanbaatar, Mongolia; m Department of Biochemistry, School of Biomedicine, Mongolian National University of Medical Sciences, Ulaanbaatar, Mongolia; n Regional Office for the Western Pacific, World Health Organization, Manila, Philippines; o World Health Organization, Geneva, Switzerland; p Victorian Infectious Diseases Reference Laboratory at the Peter Doherty Institute for Infection and Immunity, Royal Melbourne Hospital, Australia; q Department of International Cyber Education, Graduate School, Mongolian National University of Medical Sciences, Ulaanbaatar, Mongolia

**Keywords:** COVID-19, SARS-CoV-2, Immunity, Mongolia, Epidemiology, Pandemics, Seroepidemiologic studies, Seroprevalence

## Abstract

**Background:**

The COVID-19 pandemic has global impacts but is relatively understudied in developing countries. Mongolia, a lower-middle-income country, instituted strict control measures in early 2020 and avoided widespread transmission until vaccines became available in February, 2021. Mongolia achieved its 60% vaccination coverage goal by July 2021. We investigated the distribution and determinants of SARS-CoV-2 seroprevalence in Mongolia over 2020 and 2021.

**Methods:**

We performed a longitudinal seroepidemiologic study aligned with WHO's Unity Studies protocols. We collected data from a panel of 5000 individuals in four rounds between October 2020 and December 2021. We selected participants through local health centres across Mongolia by age-stratified multi-stage cluster sampling. We tested serum for the presence of total antibodies against SARS-CoV-2 receptor-binding domain, and levels of anti-SARS-CoV-2 spike IgG and neutralising antibodies. We linked participant data with national mortality, COVID-19 case, and vaccination registries. We estimated population seroprevalence and vaccine uptake, as well as unvaccinated population prior-infection prevalence.

**Findings:**

At the final round in late 2021, 82% (n = 4088) of participants completed follow-up. Estimated seroprevalence increased from 1.5% (95% CI: 1.2–2.0), to 82.3% (95% CI: 79.5–84.8) between late-2020 and late-2021. At the final round an estimated 62.4% (95% CI: 60.2–64.5) of the population were vaccinated, and of the unvaccinated population 64.5% (95% CI: 59.7–69.0) had been infected. Cumulative case ascertainment in the unvaccinated was 22.8% (95% CI: 19.1%–26.9%) and the overall infection-fatality ratio was 0.100% (95% CI: 0.088–0.124). Health workers had higher odds for being COVID-19 confirmed cases at all rounds. Males (1.72 (95% CI: 1.33–2.22)) and adults aged 20 and above (12.70 (95% CI: 8.14–20.26)) had higher odds for seroconverting by mid-2021. Among the seropositive, 87.1% (95% CI: 82.3%–90.8%) had SARS-CoV-2 neutralising antibodies by late 2021.

**Interpretation:**

Our study enabled tracking of SARS-CoV-2 serological markers in the Mongolian population over one year. We found low SARS-CoV-2 seroprevalence in 2020 and early 2021, with seropositivity increasing over a 3-month interval in 2021 due to vaccine roll out and rapid infection of most of the unvaccinated population. Despite high seroprevalence in Mongolia amongst both vaccinated and unvaccinated individuals by end-2021, the SARS-CoV-2 Omicron immune escape variant caused a substantial epidemic.

**Funding:**

10.13039/100004423World Health Organization, WHO UNITY Studies initiative, with funding by the COVID-19 Solidarity Response Fund and the German Federal Ministry of Health (BMG) COVID-19 Research and development. The Ministry of Health, Mongolia partially funded this study.

**Research in context**

**Evidence before this study**

We searched the PubMed database using ((“COVID-19” OR “SARS-CoV-2”) AND (“Seroepidemiologic Studies”)) MeSH terms. There were 1529 studies as of March 2, 2023. In addition, we searched for ((“COVID-19” OR “SARS-CoV-2”) AND (“Mongolia”)) terms and found 192 entries.

Additionally, we searched from international dashboard http://www.serotracker.com a total of 4102 seroprevalence studies in 141 countries and territories including 33,236,125 participants were notified as of November 14, 2022.

Mongolia reported one nationwide investigation from 2021 prior to COVID-19 vaccination.

**Added value of this study**

Our investigation was the second national population serological survey to be reported, as a continuation of the WHO's Unity Early epidemiological studies initiative. We covered the ongoing pandemic during the early COVID-19 vaccine roll out, demonstrating a successful vaccination campaign in a lower-middle income country. This study has been the first longitudinal study in the region as of August 2022, allowing documentation of SARS-CoV-2 infection and vaccination progress.

In the Western pacific region, several dozen studies were reported with serological samples collected before 2022, prior to the SARS-CoV-2 Omicron variant dominance. However, these studies were mostly local, blood donor, contacts, and healthcare workers; not nationally representative samples. Our study has been the only study with nationally representative samples covering Delta variant dominating timeline in the region and country. We collected serum samples after the initial national vaccination agenda completion date in mid-2021, thus providing valuable information on vaccination outcomes.

**Implications of all the available evidence**

As a longitudinal cohort with samples from the general public, this WHO Unity Early Epidemiological Study allowed tracking of infection and vaccination progress in a lower-middle income country, demonstrating the value of standardised early epidemiological investigations. Additional to the serological investigations, our study was strengthened with various national databases in COVID-19. We found similarly proportionate rates for COVID-19 deaths, confirmed cases, and vaccination. Thus, our investigation demonstrates nationally representative outcomes in Mongolia.

## Introduction

The COVID-19 pandemic had high disease burden and impact on all sectors across the globe, since it was first detected. Globally, over 6.6 million deaths and 650 million cases are confirmed as of December 2022.^1^ Seroprevalence studies of SARS-CoV-2 notify under-detected morbidity and vaccination progress, particularly in low- and middle-income countries.^2^

Mongolia, a lower-middle income country, instituted stringent public health measures early in the pandemic.^3^ Throughout 2020, Mongolia implemented border closures, educational facility closures, bans on public gatherings, mandatory mask-wearing, extensive contract tracing, quarantines and lockdowns.^3^ The first few domestic cases of COVID-19 were reported in November 2020.^4^ Subsequently, the country used RT-PCR testing extensively—from 1000 to 4000 tests per case (as 7-day rolling average) in late-2020, gradually reducing test numbers to approximately 500 tests per case in March 2021.^1^ These measures appeared successful in limiting transmission, with ∼1% population seroprevalence for SARS-CoV-2, at the first national survey in late 2020.^4^

Mongolia began their vaccine rollout in February 2021 and achieved the target coverage of at least 60% of the total population by mid-2021,^1^ initially vaccinating adults and later children under 18.^5^ Mongolia vaccinated with a combination of inactivated (the most highly distributed), adenovirus vectored, and messenger RNA SARS-CoV-2 vaccines.^5^

Coinciding with the start of the vaccine rollout, Mongolia experienced an upsurge in cases resulting in reintroduction of restrictions in late March 2021. Subsequent relaxation of restrictions in June 2021 was followed by a large epidemic associated with the Delta variant, stretching testing capacity while a substantial proportion of the population remained unvaccinated.

Serosurveys can be used to understand variation in population exposure to SARS-CoV-2 infection and vaccines by demographic groups, space, and time.^6^ In estimating prior-infection with SARS-CoV-2 they avoid the key problem of variable ascertainment due to testing access and utilization that is inherent in the use of case notification data. Longitudinal studies involving repeated sampling of the same individuals can identify exposure within bounded intervals, allowing assessment of changing patterns of primary exposure. Comparison of infection estimates from serology and case notification can then inform assessment of testing and control strategies. Nevertheless, most population SARS-CoV-2 serology studies to-date have employed single cross-sectional designs, been of limited quality, and used sampling strategies likely to give rise to biased population estimates.^6^

We used serology (infection or vaccination non-differentiating) in a large prospective population representative cohort linked to national vaccination and testing registries to investigate variation in exposure to SARS-CoV-2 infection and vaccination in Mongolia between late 2020 and late 2021, a period during which Mongolia implemented its SARS-CoV-2 vaccination program and recorded its first sustained epidemics of COVID-19.

## Methods

### Sampling and recruitment

Design, sampling and recruitment for the initial survey round are previously described in detail.^4^ The country was divided into strata consisting of the capital city (Ulaanbaatar) and four regions. Within each region two or three aimags (provinces) were selected with equal probability. Within Ulaanbaatar, the six central districts were selected with certainty. The remaining three outer districts of Ulaanbaatar were excluded from selection due lower population size and semiurban population. In each central district of Ulaanbaatar three-to-five khoroos (subdistricts) were selected with equal probability. Among aimags, the central soum or aimag centre (secondary administrative subdivisions outside Ulaanbaatar) was selected with certainty and two of the remaining soums were selected with equal probability. Finally, clusters of 100 participants were recruited through local health centres in each of the 50 selected soums or khoroos. Recruitment of participants within each soum or khoroo was stratified by age group, reflecting the 2018 Mongolian population age distribution. Initial recruitment took place in October–December 2020.

### Data collection

**Fig. 1:**
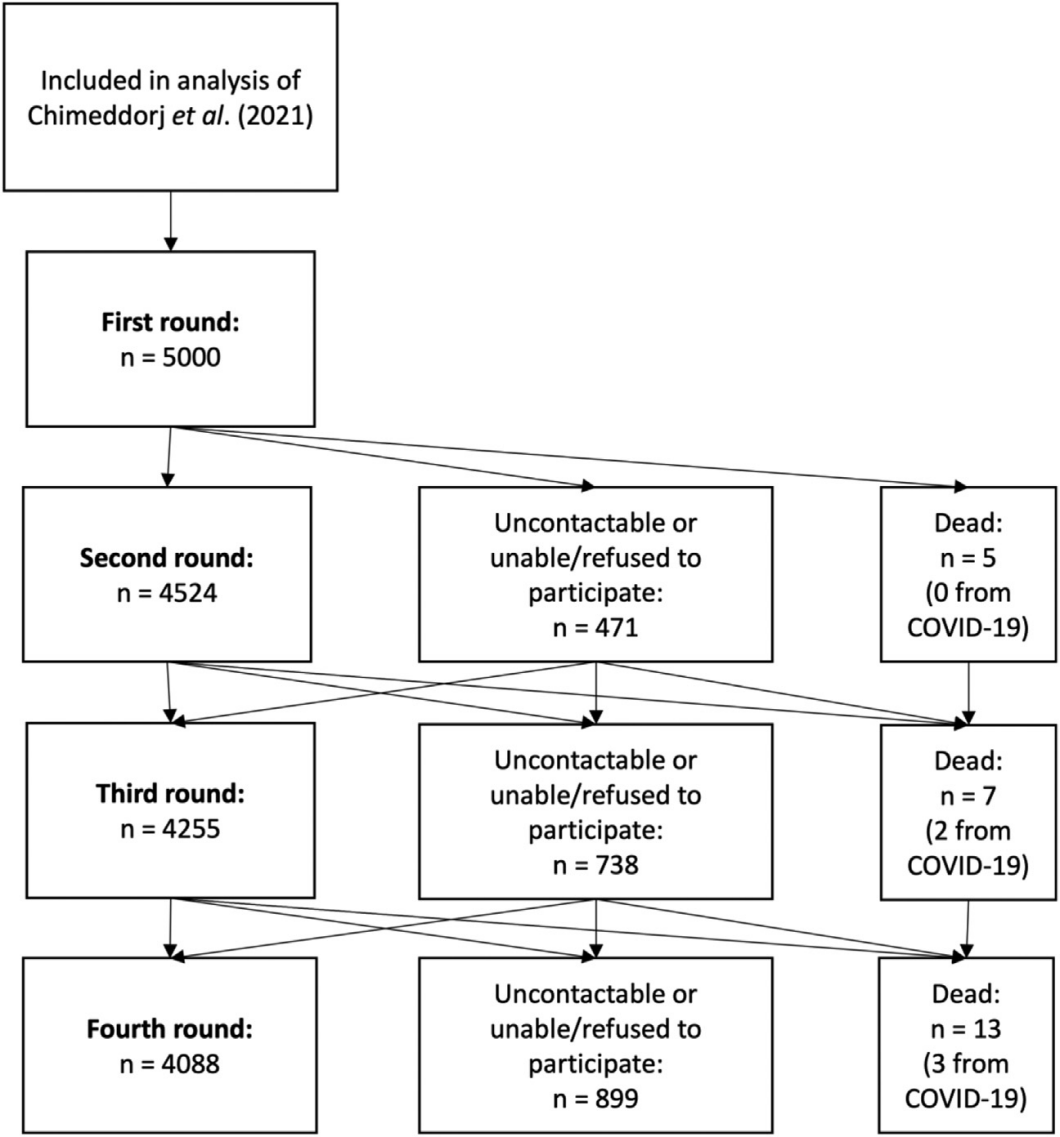
Eligibility for analysis and reasons for nonresponse at follow-up.

A detailed data collection protocol was reported previously,^4^ and we followed WHO UNITY Studies protocol for early epidemiological studies towards standardizing seroprevalences studies.^7^ Follow-up procedures included phone calls and home visits for non-responders, through the local clinics (Fig. 1). Survey instruments were adjusted at second round for introduction of nationwide SARS-CoV-2 vaccination, and at fourth round for booster dosing information.

### Laboratory analysis

We tested all samples for SARS-CoV-2 total antibodies against receptor-binding domain (WS-1096, Beijing Wantai Biological Pharmacy Enterprise). Due to the limited amount of kits available, we ran quantitative SARS-CoV-2 IgG assay (Kantaro, Quantitative SARS-CoV-2 IgG Antibody RUO Kit) tests on selected seropositive individuals to investigate antibody kinetics with intention to test all available stored seropositive samples from previous rounds. Additionally, we tested total antibody positive samples for SARS-CoV-2 neutralising antibodies (NAbs) using the Wantai SARS-CoV-2 NAbs ELISA kit (WS-1596, Beijing Wantai Biological Pharmacy Enterprise) at the third and fourth rounds.

### Linkage to national registries

We retrieved participant vaccination data including type, dose, and dates, and RT-PCR testing and rapid antigen testing results through direct linkage using national registration IDs from Ministry of Health's Gerege digital database system and the National Centre for Communicable Diseases (NCCD, approval on April 21, 2022, #21) SARS-CoV-2 nationwide registry for case confirmation, across the survey timeline with formal permission where appropriate. We searched participants missing at the fourth round from the national cause of death registry database from Centre for Health Development (approval on April 27, 2022, #37), to identify deaths and their causes that occurred during the study timeline following to completion of data collection. A total of 15 individuals were not retrievable for database searches due to incorrect registration ID numbers.

### Population-level data

We obtained cumulative counts of COVID-19 cases and deaths by age, as well as weekly proportions of circulating SARS-CoV-2 lineages and variants of concern among sequenced samples, courtesy of the NCCD. Additionally, we retrieved publicly available data on daily confirmed cases and people vaccinated from Our World in Data^1^ (https://ourworldindata.org) on 15 March 2022. We retrieved publicly available age, sex, and administrative area population statistics for 2020 from the Mongolian Statistical Information Service (https://www.1212.mn) between March and May 2022.

### Statistical analysis

We performed all statistical analyses using R version 4.1.2.^8^ We obtained weighted population prevalence estimates and adjusted odds ratios for associations with seroconversion and confirmed COVID-19, with design-based 95% confidence intervals, by logistic regression using the *svyciprop* and *svyglm* functions from package “survey”.^9^

### Calculation of weights

We calculated sampling probabilities at recruitment for each cluster as the probability of aimag (or district) selection multiplied by the conditional probability of soum (or khoroo) selection within each aimag or district. We calculated sex-specific individual sampling probabilities by dividing the number of male or female participants within each cluster by 2020 sex-specific population totals for each soum/khoroo. We calculated sampling weights as the inverse of the product of cluster and individual sampling probabilities.

We adjusted weights for non-response at subsequent rounds by applying multivariable logistic regression models for attrition,^10^ using predictor variables that were significantly unbalanced between respondents and non-respondents (Table S1). To reduce contribution to variance from extreme weights generated by this procedure, we trimmed weights above 5 times the median adjusted weight at each round, redistributing excess weights among the remaining observations.^11^ Finally, we adjusted weights at each round to align sample margins with joint age-sex, and marginal regional population distributions by iterative proportional fitting using the *rake* function.^9^

### Population seroprevalence and vaccination uptake

We defined seropositivity according to results of the qualitative Wantai total antibody assay. We estimated weighted seroprevalence at each round overall, and by age group, sex, ethnicity, region, and district of central Ulaanbaatar. We estimated seroprevalence by occupation (healthcare worker vs other), restricting analysis to working age-groups (20–59 years). We estimated vaccination uptake, defined as at least one dose of a COVID-19 vaccine recorded in the national registry, for the same subgroups.

### Prior-infection prevalence in the unvaccinated population

We defined infected individuals among the unvaccinated population at each round. We defined unvaccinated individuals as those who had no vaccinations recorded in the national registry prior to the sampling date. To reduce false negative results because of antibody decay in estimating prevalence of prior infection, and because few validation studies assess sensitivity more than 6 months after infection (Table S1), we defined previously infected individuals as those who were seropositive at the current round or any prior round (cumulative seropositivity). We adjusted prevalence estimates and 95% confidence bounds based on the manufacturer reported test specificity of 99% and sensitivity of 99% (>14 days post symptom onset) for PCR-confirmed infection^12^ according to the method described by Rogan and Gladen,^13^ truncating adjusted confidence intervals at zero or one.

### Sensitivity analyses

Acknowledging that test performance in the target population over the study period may have differed from the manufacturer reported estimates, we conducted sensitivity analyses using combinations of the highest and lowest sensitivity (overall or ≥14 days post-onset) and specificity estimates from a further 12 validation studies (Table S2). Next, we repeated these analyses using either self-report or a combination of self-report and the registry to define vaccination status (i.e. if either positive, classify as vaccinated). Although we considered registry-recorded vaccination status to be more reliable than self-report, some vaccinations may not have been recorded in the registry. Finally, we restricted the definition of infection to current, rather than cumulative, seropositivity.

### Proportion of the seropositive population with evidence of neutralising antibodies

We estimated the proportion of the seropositive population with NAbs at the third and fourth rounds, as evidenced by a binding inhibition rate of ≥50% on the Wantai SARS-CoV-2 NAbs ELISA. We obtained estimates for the same subgroups as for the seroprevalence estimates.

### Case ascertainment in the unvaccinated population

We estimated cumulative case ascertainment at the fourth study round as the proportion of ever-seropositive unvaccinated participants who had become a confirmed case, based on self-report or registry record. We obtained age-group specific and region-specific estimates.

### Infection-fatality ratio

We estimated overall and age-specific cumulative infections at the fourth round by dividing cumulative numbers of nationally reported cases as of the median date of follow-up for the fourth round (13 September 2021) by our case ascertainment estimates for the unvaccinated population. We then estimated infection-fatality ratios (IFR) by dividing estimated numbers of infections by cumulative nationally reported COVID-19 deaths as of 11 October 2021 (allowing for a four-week lag between seroconversion and death). Thus, we assumed that the probability of any infected individual becoming a case was independent of vaccination status.

### Symptomatic fraction in the unvaccinated population

At each round, we considered seropositive unvaccinated participants who were seronegative at the previous round (new-seropositive), to have been infected since the previous round. Among this subgroup, we estimated the prevalence of COVID-19 compatible symptoms in the prior three months (Table S3), overall and by age group. We obtained estimates for two definitions of symptomatic: report of either at least one, or at least two symptoms. Because this analysis required classification of individual participants as infected/non-infected, and because seropositivity is an imperfect classifier of infection, we first assessed the test predictive value in identifying infected and non-infected participants, based on manufacturer reported test performance and sample prevalence of new-seropositivity at each round (Figure S1).

### Association of demographic factors with recent infection in the susceptible unvaccinated population

At each follow-up round, we estimated adjusted odds ratios for associations of sex, age group, occupation, ethnicity, region, and presence of comorbidities with new-seropositivity amongst previously seronegative unvaccinated participants. Again, we considered test predictive value in correctly classifying infection/non-infection before presenting these results.

### Association of demographic factors with recent confirmed COVID-19

At each follow-up round, we estimated adjusted odds ratios for associations of sex, age group, occupation, ethnicity, region, and presence of comorbidities with recent confirmed COVID-19. We defined recent COVID-19 as a self-reported positive test in the prior three months or having a positive COVID-19 test (RT-PCR or antigen test) recorded in the national registry since the previous survey round.

### Antibody kinetics

We assessed trends in antibody concentrations measured via the Kantaro IgG and Wantai NAbs assays in two sets of subgroups: 1) Participants who had two doses of vaccine recorded in the registry and had at least two subsequent results available without interim vaccination or confirmed infection (as defined above), with the first result obtained at least 28 days after the second dose of vaccine; and 2) Participants with confirmed infection and who had at least two subsequent results available without interim vaccination, with the first result obtained at least 28 days post positive test.

Results for the Kantaro assay were left-censored at 3.2 AU/ml and right-censored at 160 AU/ml, and for the Wantai NAbs assay at 0.0625 U/ml and 0.5 U/ml, respectively. We retested a subset of samples with previously right-censored results after dilution to obtain uncensored values (49% for Kantaro and 26% for the Wantai NAbs assay).

We fit lognormal distributions to results of both assays using *fitdistcens* in package “fitdistrplus”.^14^ We then replaced censored values by resampling from these fitted distributions, after truncating at the censoring limits. We fit separate mixed-effects linear models with random intercepts to the log-transformed data for each subgroup using package “lme4”.^15^ We performed this procedure using 1000 bootstraps then used Rubin's rules^16^ to incorporate both model and imputation related uncertainty into parameter estimates.

### Ethics statement

We obtained ethical clearances from the WHO's WPRO ethical review board (2020.9.MOG.1.ESR, October 22, 2020) and research ethical review board at the Ministry of Health, Mongolia (#193, October 1, 2020). Consent forms were introduced and obtained at the first round of the survey. At the fourth round of data collection, consent to retrieve SARS-CoV-2 case confirmation and vaccination information from national digital databases were obtained by national registration ID search.

### Role of the funding source

The funders of the study had no direct role in the study design, data collection, data analysis, data interpretation, or writing of the report.

## Results

### Round timing and epidemic context

**Fig. 2:**
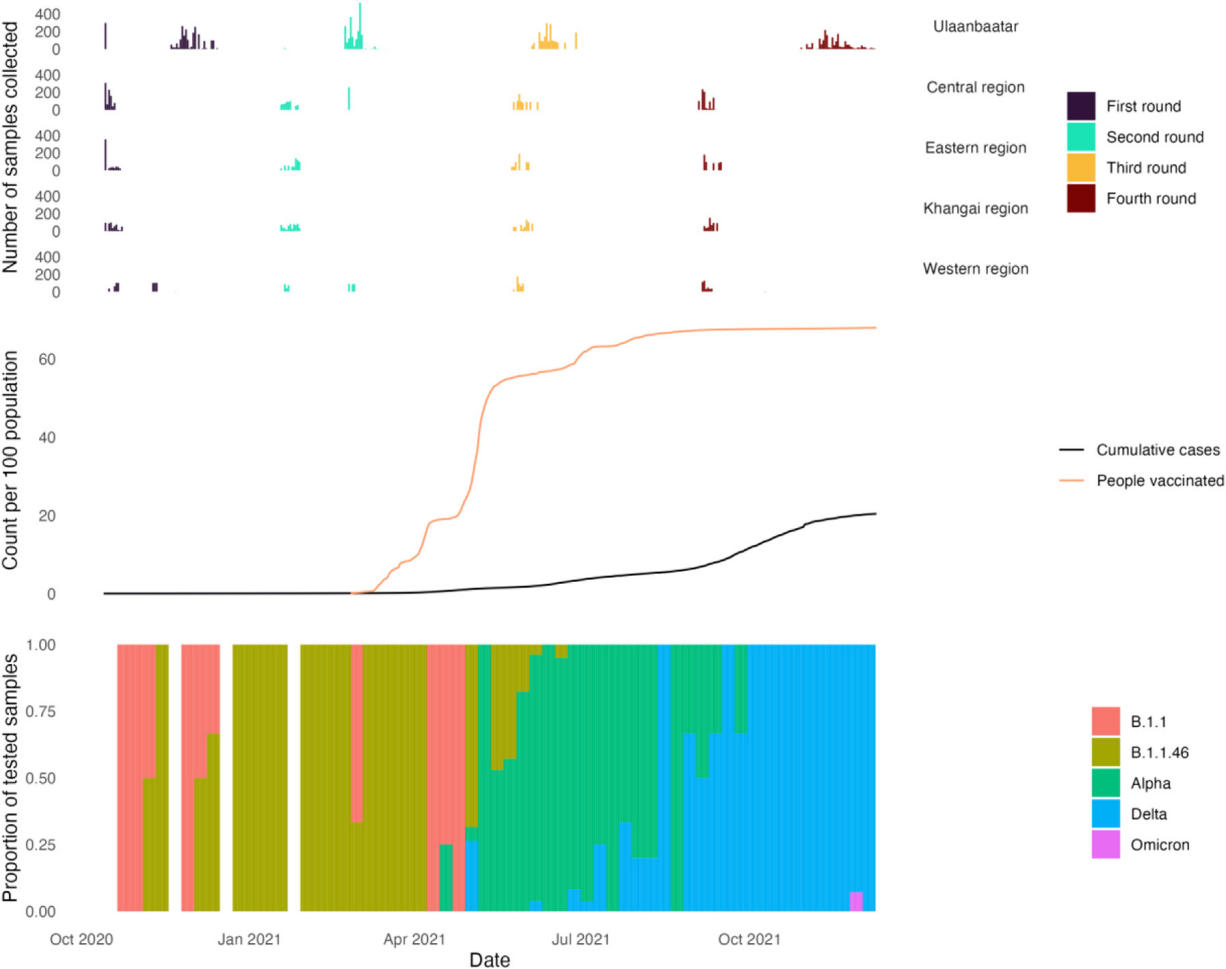
Epidemic context top: daily number of samples collected by region and survey round; middle: cumulative cases and people vaccinated; bottom: weekly lineage/variant prevalence amongst samples sequenced at the Mongolia National Centre for Communicable Diseases.

Data collection for the first survey round began on 13 October 2020 and concluded for the fourth round on 9 December 2021 (Table S4). The bulk of samples were collected at approximately three-month intervals, however the median date of sample collection for participants in Ulaanbaatar lagged approximately 2–6 weeks behind the regions for the first, second, and third rounds, and approximately 10 weeks behind for the fourth round (Fig. 2).

Collection of data for the first and second rounds preceded sustained reported community transmission (Fig. 2). The national vaccination rollout began on 23 February, roughly coinciding with completion of data collection for the second round. By the third round, approximately 50–60% of the population had received at least one dose of vaccine, and the country was experiencing sustained transmission of the Alpha variant. By September 2021, whole population vaccine uptake plateaued at around 67–68%. The fourth round coincided with the Delta variant becoming dominant amongst sequenced samples, and a rapid rise in cumulative confirmed cases to 20.4 per 100 population by the end of the study.

### Sample characteristics

At recruitment, the median age of participants was 27 years (range 0–90 years), and 65% were female (Table 1). Participants recruited from Ulaanbaatar accounted for 46% of the sample and 22% reported at least one medical comorbidity.

**Table 1:** Participant characteristics by survey round (unweighted).

	First round (N = 5000)	Second round (N = 4524)	Third round (N = 4255)	Fourth round (N = 4088)
**Sex**				
Female	3242 (64.8%)	2958 (65.4%)	2799 (65.8%)	2701 (66.1%)
Male	1758 (35.2%)	1566 (34.6%)	1456 (34.2%)	1387 (33.9%)
**Age group (years)**				
0–4	580 (11.6%)	356 (7.9%)	335 (7.9%)	192 (4.7%)
5–9	562 (11.2%)	545 (12.0%)	494 (11.6%)	504 (12.3%)
10–14	430 (8.6%)	420 (9.3%)	391 (9.2%)	398 (9.7%)
15–19	328 (6.6%)	287 (6.3%)	270 (6.3%)	294 (7.2%)
20–29	815 (16.3%)	692 (15.3%)	638 (15.0%)	558 (13.6%)
30–39	823 (16.5%)	776 (17.2%)	726 (17.1%)	740 (18.1%)
40–49	677 (13.5%)	646 (14.3%)	609 (14.3%)	590 (14.4%)
50–59	468 (9.4%)	483 (10.7%)	484 (11.4%)	477 (11.7%)
60–69	218 (4.4%)	215 (4.8%)	211 (5.0%)	231 (5.7%)
70+	99 (2.0%)	104 (2.3%)	97 (2.3%)	104 (2.5%)
**Region**				
Ulaanbaatar	2300 (46.0%)	2072 (45.8%)	1905 (44.8%)	1807 (44.2%)
Central region	900 (18.0%)	840 (18.6%)	820 (19.3%)	827 (20.2%)
Eastern region	600 (12.0%)	573 (12.7%)	550 (12.9%)	538 (13.2%)
Khangai region	600 (12.0%)	573 (12.7%)	547 (12.9%)	532 (13.0%)
Western region	600 (12.0%)	466 (10.3%)	433 (10.2%)	384 (9.4%)
**Ethnicity**				
Khalkh	4073 (81.5%)	3732 (82.5%)	3527 (82.9%)	3395 (83.0%)
Kazakh	315 (6.3%)	215 (4.8%)	181 (4.3%)	159 (3.9%)
Buriad	214 (4.3%)	208 (4.6%)	205 (4.8%)	200 (4.9%)
Others	226 (4.5%)	204 (4.5%)	188 (4.4%)	186 (4.6%)
Missing	172 (3.4%)	165 (3.6%)	154 (3.6%)	148 (3.6%)
**Occupation**				
Healthcare worker	588 (11.8%)	569 (12.6%)	510 (12.0%)	471 (11.5%)
Other	4412 (88.2%)	3955 (87.4%)	3745 (88.0%)	3617 (88.5%)
**Any medical comorbidity**				
No	3695 (73.9%)	3480 (76.9%)	3242 (76.2%)	3103 (75.9%)
Yes	1093 (21.9%)	1044 (23.1%)	1006 (23.6%)	982 (24.0%)
Missing	212 (4.2%)	0 (0%)	7 (0.2%)	3 (0.1%)
**Positive COVID-19 test in prior 3 months recorded in national registry**				
No	4985 (99.7%)	4507 (99.6%)	4121 (96.9%)	3484 (85.2%)
Yes	0 (0%)	6 (0.1%)	126 (3.0%)	591 (14.5%)
Missing	15 (0.3%)	11 (0.2%)	8 (0.2%)	13 (0.3%)
**Self-reported positive COVID-19 test in prior 3 months**				
No	4904 (98.1%)	4459 (98.6%)	3668 (86.2%)	3279 (80.2%)
Yes	69 (1.4%)	56 (1.2%)	106 (2.5%)	701 (17.1%)
Unknown	26 (0.5%)	9 (0.2%)	11 (0.3%)	95 (2.3%)
Missing	1 (0.0%)	0 (0%)	470 (11.0%)	13 (0.3%)
**Self-reported or national registry recorded positive COVID-19 test in prior 3 months**				
No	4931 (98.6%)	4462 (98.6%)	4054 (95.3%)	3170 (77.5%)
Yes	69 (1.4%)	62 (1.4%)	201 (4.7%)	918 (22.5%)
**COVID-19 vaccination recorded in national registry**				
No	4985 (99.7%)	4400 (97.3%)	1929 (45.3%)	1457 (35.6%)
Yes	0 (0%)	113 (2.5%)	2318 (54.5%)	2605 (63.7%)
Missing	15 (0.3%)	11 (0.2%)	8 (0.2%)	26 (0.6%)
**Self-reported COVID-19 vaccination status**				
No	0 (0%)	1548 (34.2%)	1662 (39.1%)	1171 (28.6%)
Yes	0 (0%)	50 (1.1%)	2049 (48.2%)	2904 (71.0%)
Missing	5000 (100%)	2926 (64.7%)	544 (12.8%)	13 (0.3%)
**One or more COVID-19 compatible symptoms in prior 3 months**				
No	3654 (73.1%)	3681 (81.4%)	3117 (73.3%)	2942 (72.0%)
Yes	1289 (25.8%)	805 (17.8%)	619 (14.5%)	1073 (26.2%)
Missing	57 (1.1%)	38 (0.8%)	519 (12.2%)	73 (1.8%)
**Two or more COVID-19 compatible symptoms in prior 3 months**				
No	4024 (80.5%)	4009 (88.6%)	3306 (77.7%)	3184 (77.9%)
Yes	919 (18.4%)	477 (10.5%)	430 (10.1%)	831 (20.3%)
Missing	57 (1.1%)	38 (0.8%)	519 (12.2%)	73 (1.8%)
**Contact with a suspected or confirmed case of COVID-19 in prior 3 months**				
No	4904 (98.1%)	4459 (98.6%)	3464 (81.4%)	3033 (74.2%)
Yes	69 (1.4%)	56 (1.2%)	262 (6.2%)	876 (21.4%)
Unknown	26 (0.5%)	9 (0.2%)	59 (1.4%)	166 (4.1%)
Missing	1 (0.0%)	0 (0%)	470 (11.0%)	13 (0.3%)
**Wantai Total Ab result**				
Negative	4924 (98.5%)	4459 (98.6%)	2049 (48.2%)	736 (18.0%)
Positive	76 (1.5%)	64 (1.4%)	2206 (51.8%)	3339 (81.7%)
Missing	0 (0%)	1 (0.0%)	0 (0%)	13 (0.3%)
**Wantai NAbs ELISA result**				
Negative	0 (0%)	0 (0%)	848 (19.9%)	477 (11.7%)
Positive	0 (0%)	0 (0%)	1344 (31.6%)	2869 (70.2%)
Missing	5000 (100%)	4524 (100%)	2063 (48.5%)	742 (18.2%)

Of the 5000 participants at recruitment, 4088 (82%) participated in the fourth round. Male participants, those residing in Ulaanbaatar and Western region, and those without COVID-19 vaccination recorded in the national registry were overrepresented among non-respondents at subsequent rounds (Table S4).

### Seroprevalence, vaccination uptake, and prior-infection prevalence

**Fig. 3:**
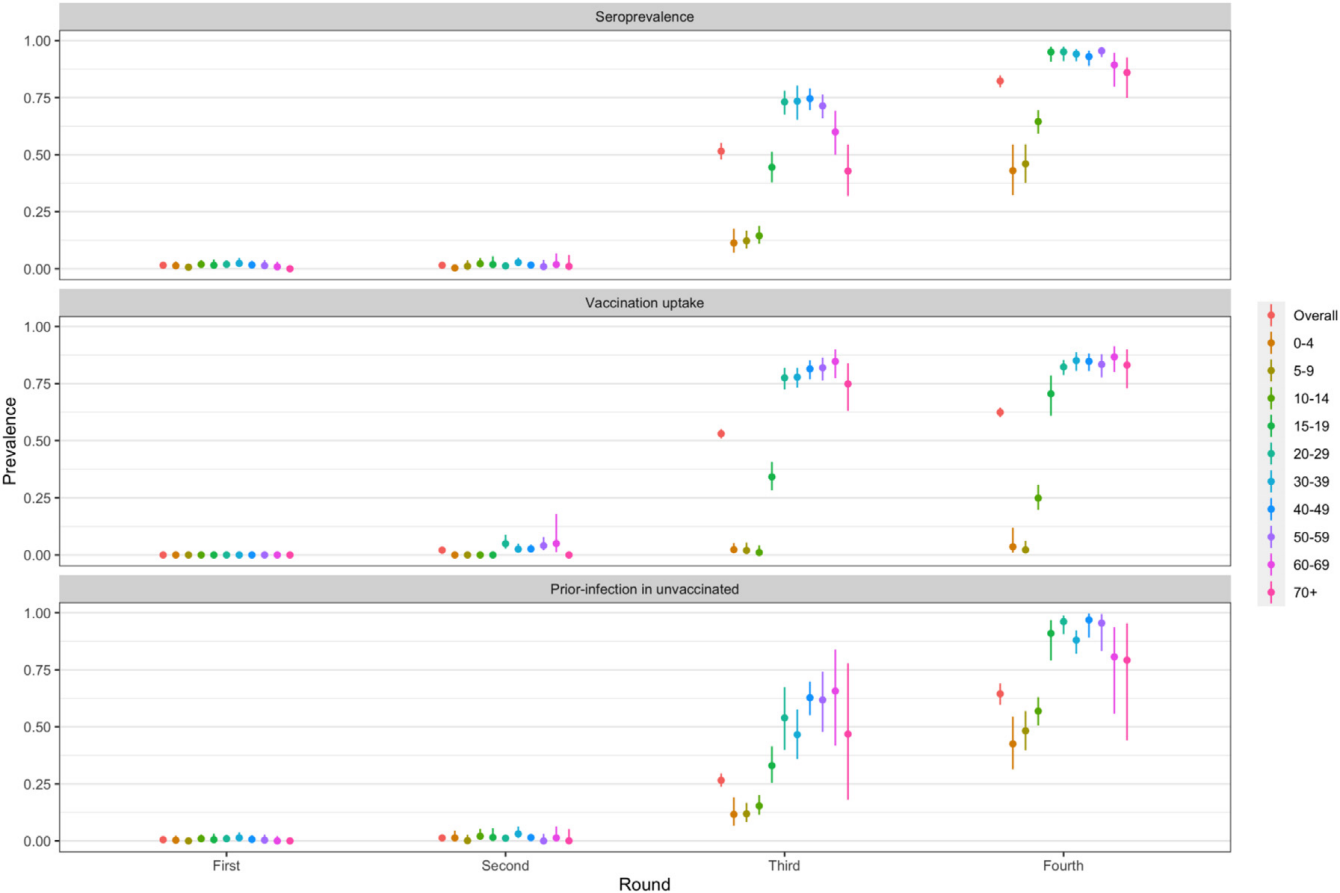
Estimated total population seroprevalence (top), vaccine uptake (middle; at least one dose), and prevalence of prior infection in the unvaccinated population (bottom) at each survey round, overall and by age group. Error bars show 95% confidence intervals.

**Fig. 4:**
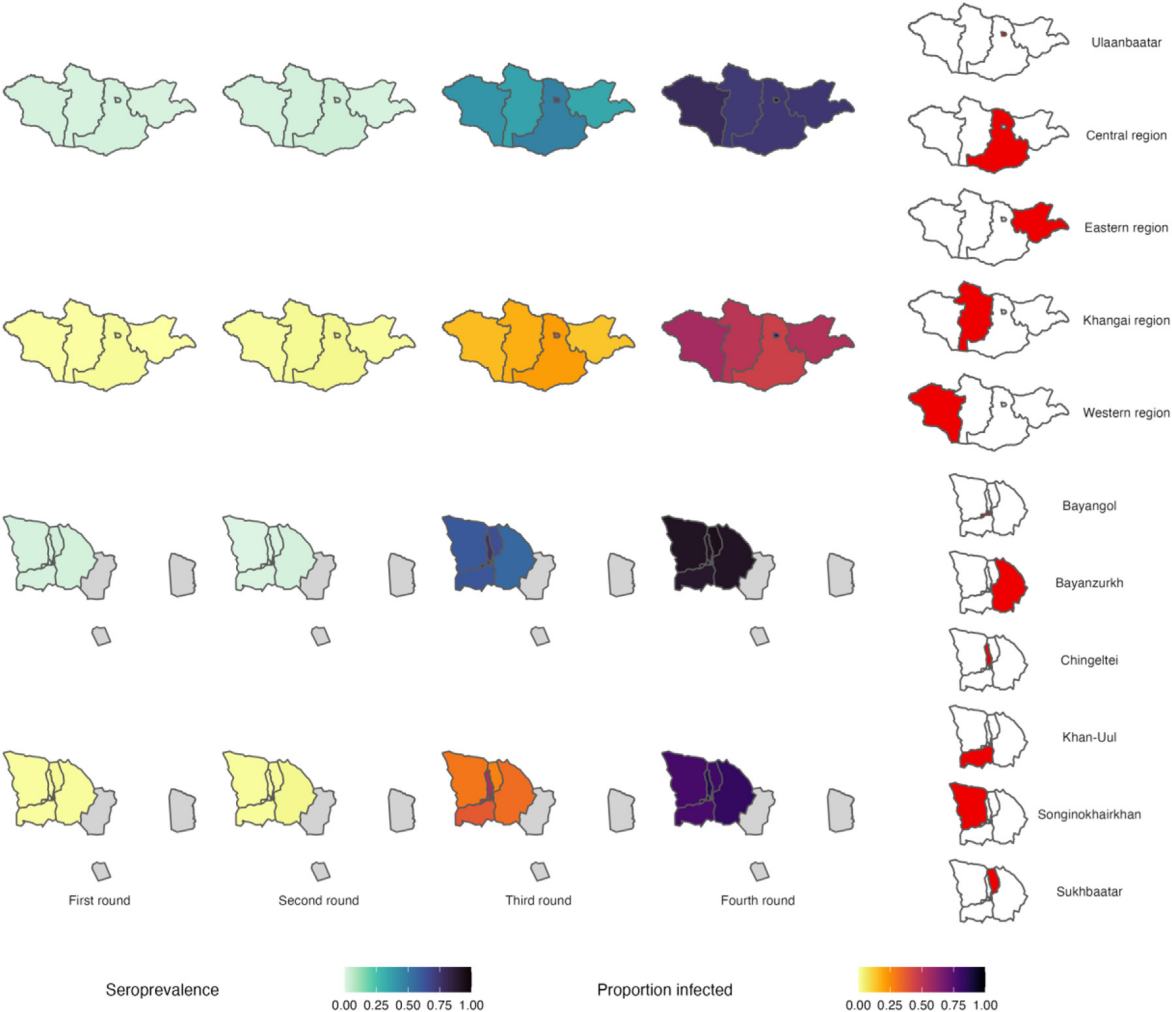
Estimated total population seroprevalence and prevalence of prior infection in the unvaccinated population at each survey round, by region (top) and district of Ulaanbaatar (bottom). Unsampled districts of Ulaanbaatar are shown in grey on district level maps, and are merged with the surrounding Central region on country maps for simplicity. Confidence intervals are presented in Tables S5 and S7. Note that samples for Ulaanbaatar were generally collected later that in the regions and are not directly comparable (see Fig. 2 and Table S4).

Estimated population seroprevalence increased from 1.5% (95% CI: 1.2–2.0%) at the first round to 82.3% (95% CI: 79.5%–84.8%) at the fourth round (Fig. 3, Table S5). At the third and fourth rounds, seroprevalence was higher in young and middle-aged adults, compared to children and older adults. Healthcare workers, compared with non-healthcare workers, had slightly higher estimated seroprevalence at the third and fourth rounds. Estimates were generally similar across regions (Fig. 4), except for Ulaanbaatar where seroprevalence was approximately 20% higher at the third and fourth rounds.

Estimated vaccine uptake was 2.1% (95% CI: 1.3%–3.5%), 53.0% (95% CI: 51.0%–55.1%), and 62.4% (95% CI: 60.2%–64.5%) at the second, third, and fourth rounds respectively (Table S6). Uptake remained low amongst under-10s at the fourth round, consistent with aged-based eligibility, while reaching >80% in all adult age groups. Uptake was slightly higher for females and similar across Ulaanbaatar and the regions.

The estimated proportion of the unvaccinated population previously infected with SARS-CoV-2 increased from 1.3% (95% CI: 0.7%–2.0%) at the second round to 26.5% (95% CI: 23.7%–29.5%) at the third round and 64.5% (95% CI: 59.7%–69.0%) at the fourth round (Table S7). Patterns in age-, sex-, and occupation-specific estimates were consistent with those for overall seroprevalence. Estimates for Ulaanbaatar were higher than for the regions by approximately 15–20% at the third round and approximately 30–35% at the fourth round.

Results of sensitivity analyses for prior-infection prevalence are provided in Figure S3. Varying sensitivity and specificity parameters made only minor differences to estimates relative to their corresponding statistical precision, except at low prevalence (i.e., the first and second rounds). When compared to registry record, self-reported vaccination status had 85% percent positive percent agreement and 78% negative percent agreement among participants for whom both were available. Defining vaccination based on self-report led to ∼10% higher estimates at the third round and ∼10% lower estimates at the final round compared to registry recorded vaccination. Defining participants as vaccinated based on either affirmative self-report or a registry recorded vaccination led to lower estimates at both rounds. Use of current instead of cumulative seropositivity to define infection did not substantially change estimates. Overall, point estimates from sensitivity analyses for the third round ranged from 16.6% to 37.4%, and for the fourth round from 48.2% to 71.3%.

### Proportion of the seropositive population with evidence of neutralising antibodies

NAb binding inhibition results were available for >99% of seropositive participants at the third and fourth rounds (Table S8). At the fourth round, the estimated proportion of seropositive individuals with a positive NAb result was 87.1% (95% CI: 82.3%–90.8%). Estimates were highest for adolescent age groups and lowest for the elderly (Table S9).

### Case ascertainment in the unvaccinated population

Estimated cumulative case ascertainment at the final study round was 22.8% (95% CI: 19.1%–26.9%; Table S10). Ascertainment was generally higher in adults (point estimates ranging from 27.2% to 44.9%) compared with child and adolescent age groups (point estimates ranging from 12.8% to 21.3%).

### Infection-fatality ratio

By 11 October 2021, a total of 1470 deaths were reported in Mongolia, with half (736) occurring in the 70+ years age group. The overall estimated IFR was 0.100% (95% CI: 0.088%–0.124%; Table S11). Estimated IFRs were low across child and adolescent age groups (∼0.001%–0.01%), then increased gradually with age to 2.139% (95% CI: 0.654%–4.615%) in the 70+ years age group.

### Symptomatic fraction in the unvaccinated population

Low estimated positive predictive values of seropositivity for infection at the first and second rounds (i.e., <25%) precluded meaningful interpretation of estimates of symptomatic fraction and association of demographic factors with seroconversion (Figure S1). The estimated symptomatic fraction was 13.6% (95% CI: 8.6%–20.8%) at the third round and 24.7% (95% CI: 18.4%–32.3%) at the fourth round based on report of at least one symptom (Table S12).

### Association of demographic factors with seroconversion in the susceptible unvaccinated population

For the third and fourth rounds respectively, higher odds of seroconversion were associated with male sex, being aged 20–59 years or 60+ relative to the baseline of 0–19 years, and residing within Ulaanbaatar (all regional estimates; Table 2).

**Table 2:** Adjusted odds ratios for association of demographic factors with seroconversion among previously seronegative unvaccinated participants.

Characteristic	Third round (n = 1762)		Fourth round (n = 952)	
	OR (95% CI)	p-value	OR (95% CI)	p-value
Sex				
Female				
Male	1.72 (1.33–2.22)	<0.01	1.40 (1.04–1.89)	0.03
Age group (years)				
0–19				
20–59	12.70 (8.14–20.26)	<0.01	9.28 (6.06–14.63)	<0.01
60+	10.28 (5.43–19.78)	<0.01	3.96 (1.51–10.96)	<0.01
Ethnicity				
Khalkh				
Kazakh	0.85 (0.37–2.00)	0.70	3.05 (1.68–5.63)	<0.01
Buriad	0.58 (0.08–3.07)	0.55	0.98 (0.43–2.19)	0.95
Others	0.63 (0.37–1.04)	0.08	1.00 (0.30–3.34)	0.99
Region				
Ulaanbaatar				
Central	0.31 (0.13–0.67)	<0.01	0.14 (0.09–0.23)	<0.01
Eastern	0.16 (0.08–0.30)	<0.01	0.23 (0.17–0.31)	<0.01
Khangai	0.19 (0.12–0.30)	<0.01	0.18 (0.12–0.25)	<0.01
Western	0.12 (0.06–0.23)	<0.01	0.16 (0.10–0.27)	<0.01
Occupation				
Healthcare worker				
Other	0.94 (0.63–1.44)	0.79	0.82 (0.42–1.60)	0.57
Any comorbidity				
No				
Yes	1.11 (0.72–1.70)	0.64	0.76 (0.43–1.35)	0.36

OR = Odds Ratio, CI = Confidence Interval.

### Association of demographic factors with confirmed COVID-19

Similar demographic associations observed for seroconversion were also observed for confirmed COVID-19, although there were no consistent sex-based associations, and age-based effects were considerably smaller (Table 3). Being a non-healthcare worker was associated with lower odds of confirmed COVID-19 at all rounds.

**Table 3:** Adjusted odds ratios for association of demographic factors with first diagnosis of confirmed COVID-19.

Characteristic	First round (n = 4621)		Second round (n = 4359)		Third round (n = 4094)		Fourth round (n = 3937)	
	OR (95% CI)	p-value	OR (95% CI)	p-value	OR (95% CI)	p-value	OR (95% CI)	p-value
Sex								
Female								
Male	0.92 (0.48–1.75)	0.82	1.06 (0.48–2.29)	0.88	1.02 (0.75–1.39)	0.90	0.80 (0.67–0.95)	0.01
Age group (years)								
0–19								
20–59	1.40 (0.71–2.84)	0.37	2.58 (0.95–8.58)	0.09	1.75 (1.17–2.66)	0.01	3.30 (2.41–4.58)	<0.01
60+	1.18 (0.19–4.82)	0.84	3.00 (0.33–23.67)	0.29	1.92 (0.92–3.81)	0.09	2.30 (1.59–3.32)	<0.01
Ethnicity								
Khalkh								
Kazakh	2.12 (0.64–6.81)	0.24	0.00 (0.00- >99)	0.99	0.33 (0.03–1.42)	0.25	0.98 (0.47–2.03)	0.95
Buriad	0.24 (0.04–0.78)	0.06	0.40 (0.06–1.44)	0.25	0.23 (0.06–0.64)	0.02	0.70 (0.35–1.30)	0.30
Others	0.48 (0.12–1.28)	0.24	2.76 (1.03–6.35)	0.03	1.23 (0.67–2.10)	0.49	1.67 (1.05–2.64)	0.04
Region								
Ulaanbaatar								
Central	0.04 (0.00–0.20)	<0.01	1.11 (0.37–2.92)	0.85	0.22 (0.06–0.60)	0.02	0.17 (0.06–0.41)	<0.01
Eastern	0.72 (0.20–1.88)	0.57	0.38 (0.08–1.11)	0.14	0.64 (0.30–1.22)	0.23	0.47 (0.36–0.60)	<0.01
Khangai	0.50 (0.07–2.01)	0.41	0.59 (0.09–2.50)	0.53	0.23 (0.08–0.52)	<0.01	0.18 (0.12–0.28)	<0.01
Western	0.46 (0.15–1.23)	0.18	0.10 (0.01–0.38)	0.01	0.05 (0.01–0.16)	<0.01	0.39 (0.25–0.58)	<0.01
Occupation								
Healthcare worker								
Other	0.36 (0.19–0.70)	<0.01	0.10 (0.03–0.32)	<0.01	0.31 (0.20–0.48)	<0.01	0.70 (0.50–1.00)	0.06
Any comorbidity								
No								
Yes	1.01 (0.33–2.62)	0.99	1.27 (0.55–2.74)	0.56	1.21 (0.86–1.69)	0.29	1.38 (1.11–1.70)	<0.01

OR = Odds Ratio, CI = Confidence Interval.

### Antibody kinetics

There was substantial intra-individual heterogeneity in both post-vaccination and post-infection antibody concentrations and trends for both assays (Figure S3). Overall, no population level decline in antibody level after either infection or vaccination was evident over the study period. Characteristics of participants included in analysis of antibody kinetics are given in Table S13 and S14.

## Discussion

We conducted a longitudinal, age-stratified, population-based seroepidemiological investigation aligned with WHO's UNITY protocols^7^ spanning 12 months of the COVID-19 pandemic in Mongolia across 2020 and 2021. We found low SARS-CoV-2 seroprevalence in 2020 and early 2021, increasing to 51.5% (47.8–55.2) in May–June 2021 and 82.3% (79.5–84.8) in September–December 2021. We supplemented survey data with national registry and population-level data including all-cause mortality, COVID-19 case confirmation, and vaccination data, providing additional robustness and context.

Mongolia maintained stringent policies to contain COVID-19 throughout 2020 and the first half of 2021, and gradually reduced policy measures after introducing vaccines in February 2021.^3^ Early policy actions and pandemic response taken by Mongolia were strong among lower- and middle-income countries.^3^ First and second round seroprevalence show that these policy measures suppressed transmission in the population until vaccines became available, whereas third and fourth round seroprevalence rates are high, likely due to a combination of vaccination and SARS-CoV-2 infection.

Seroprevalence remained at approximately 1% among the general public until early 2021 in Mongolia, before vaccine roll out. Across regions, the Western Pacific had the lowest prevalence ratios in pre-vaccination period compared to Americas.^17^ Meanwhile, nearby countries including Kazakhstan had much higher seroprevalence of 56.7% in urban locations at a similar time in 2021, potentially due to having less stringent public health measures before vaccines became available.^18^ A supporting global estimate shows the COVID-19 cumulative infection ratio and IFRs were lower in Mongolia compared to the region of central Asia and eastern Europe, when pandemic preparedness and risk communication were reflected by health promotion, as a mean of disease containment.^19^

In September–December 2021, overlapping with the Delta variant predominant period, our findings suggest that 82.3% (95% CI: 79.5–84.8) of the Mongolian population were seropositive and 62.4% (95% CI: 60.2–64.5) were vaccinated. A study in Gauteng province of South Africa surveyed seroprevalences in large samples (n = 7010) from general public between October 22 and December 9, 2021 preceding the Omicron wave.^20^ Overall, 73.1% (95% CI: 72.0–74.1) seroprevalence was observed, whereas only 18.8% had COVID-19 vaccinations.^20^ Both studies show high seroprevalence rates at pre-Omicron stage, despite large differences in vaccination coverage.

Vaccine equity has been a global health priority set by the WHO and other international communities during the COVID-19 pandemic response, particularly towards low and lower-middle-income countries.^21^ Vietnam, a regional country with similar GDP per capita to Mongolia, reported 42.6% overall seropositivity and 13% vaccination at least 14 days prior among the close contacts of confirmed cases during September–October, 2021 in study conducted in Ho Chi Minh city.^22^ Meanwhile, our findings showed 92.2% (95% CI: 90.1–93.8) seropositivity rate and 63.9% (95% CI: 60.6–67.0) vaccinated in Ulaanbaatar city, indicating higher vaccine roll-out at a similar time. In June–July 2021, around 60% of India's general population were seropositive and only 24.8% had at least one dose of COVID-19 vaccination. In contrast, despite the enormous population size differences, our survey found near 52% seropositive and near half of participants were vaccinated at a similar time.^23^ When compared with both studies in Viet Nam^22^ and India,^23^ Mongolia had rolled out COVID-19 vaccination at a much faster pace. The majority of both the general public and healthcare workers were accepting of COVID-19 vaccination in Mongolia throughout the vaccine roll out period.^24^^,^^25^ In addition, Mongolia achieved its 60% population coverage vaccination goal with at least a single dose by July 1st 2021 predominantly by inactivated SARS-CoV-2 vaccines, and later boosted by mRNA or adenoviral vector vaccines, well prior to global rates (Jan 20, 2022) and lower-and middle-income (April 30, 2022) countries.^1^ Similar epidemic characteristics can be experienced where the population is vaccinated by inactivated SARS-CoV-2 vaccines, including regional country of the PRChina.

Our study found differences in seroprevalence by age group, as expected. Children and adolescents aged under 15 years had considerably lower seropositivity than adults. Among children, Mongolia targeted initially those aged 16 and above (Order A/368, June 15, 2021), followed by 12 and above (Order A/404, June 23, 2021), and lastly 5–11 year olds (Order A/378, July 20, 2022), for COVID-19 vaccination by mRNA vaccine.^26^ Children and adolescents under 20 had lower odds of being COVID-19 cases than adults by the end of the survey. Coupled with the return of fully in-class educational services from September 1, 2021, we expect childhood morbidity to escalate in Mongolia.^27^ Paediatric hospitalisations subsequently increased during the Omicron wave compared with previous waves.^28^

Based on extrapolation of estimated case-ascertainment from the unvaccinated population, around 40% of Mongolians had been infected by late 2021 (Table S11). Mongolia confirmed 680,000 (21% of population) cumulative cases to December 10, 2021, despite considerable under reporting of cases due to limited testing availability.^1^ Relative to population size, Mongolia had much higher cumulative reported cases by late 2021 than the regional countries including Viet Nam, South Korea, and Australia, despite being the most dispersedly populated country.^1^

Ulaanbaatar city had much higher prior infection rates in the survey data which was collected approximately 4–8 weeks after rural samples. Despite being sampled earlier, the rural population also had a considerable amount of previous SARS-CoV-2 infection. Ulaanbaatar is the only major metropolitan area in Mongolia and hosts centralised government facilities located in the capital, reflecting the population connectedness to the city, despite being the most dispersedly populated country in the world. Similarly, a large-scale United States cohort showed no difference over the infection between rural and urban regions as the COVID-19 pandemic progressed.^29^

We observed several sociodemographic factors associated with being a first-time confirmed case of COVID-19 across study period. Residing in the four rural regions of Mongolia was associated with a lower odds of being a confirmed case relative to Ulaanbaatar city in the second half of 2021, with the only exception being the eastern region in the third survey round. A large-scale cohort study in the United States showed living in urban areas was associated with higher risk of having SARS-CoV-2 infection earlier, whilst these associations disappeared 9 months later in November 2020 by which time infection was widespread.^29^ The rural population remained at lower risks of SARS-CoV-2 infection in 2021, potentially due to nomadic herding lifestyle, lower population density, and smaller household sizes than the Ulaanbaatar city.^30^ Non-healthcare professions were associated with lower risks of being a COVID-19 confirmed case compared to healthcare workers consistently throughout the four rounds of the survey in 2020 and 2021. Various studies found frontline healthcare workers had higher risks for COVID-19 infection than the general public,^31^ as well as occupational risks including lack of PPE, intubating patients, and being female.^32^ At the fourth round during late 2021, males had nearly 20% lower odds than females of being a confirmed case. Subjects with any of the comorbidities had 40% higher odds of being a confirmed case by late 2021. However, this association may be a result of increased severity of disease amongst those with comorbidities resulting in hospitalisation and thus greater ascertainment.^33^

Humoral immunity plays an essential role in SARS-CoV-2 infection protection, preferably determined by neutralising antibodies.^34^ Participants had high positive rates of NAbs against SARS-CoV-2 (Table S13) by end 2021, whilst available follow-up showed growing pattern in anti-SARS-CoV-2 IgG titres. Furthermore, current SARS-CoV-2 NAb titre tests were designed towards ancestral antigens namely spike.^35^

Our study encountered three COVID-19 deaths in a cohort of 5000 (60.0 per 100,000) among subjects followed in 2020 and 2021, over a year timeline. Mongolia confirmed 1986 deaths (59.15 per 100,000 for 2020 population size 3,357,542) as of January 1, 2022.^36^ Quality and timeliness of death registry data remain a challenging issue globally, hindering equitable comparison particularly for COVID-19.^37^ According to global estimates, rates of excess death and COVID-19 mortality in Mongolia were half of those observed in the broader Central Asia region by end 2021.^38^ These could reflect effectiveness of rapid vaccine rollout towards preventing the severe outcomes. The IFR in our study may not be representative of the general population, since estimates was based on the unvaccinated study participants, who may also have different health seeking behaviour and exposure history.

Following our study, Mongolia experienced a substantial epidemic in early 2022 consisting of predominantly the Omicron variant.^1^^,^^36^ Despite the fact that confirmed cases between January and August of 2022 comprised nearly one-third of all COVID-19 cases in Mongolia, the highest recorded weekly moving average of deaths due COVID-19 was low at 3.00 (Feb 15, 2022) compared to 18.14 (October 3, 2021) in the peak of Delta variant predominant period.^1^^,^^36^ When considered relative to population size, the highest recorded weekly moving average of COVID-19 deaths in Mongolia was 0.90, lower than the global mean of 1.39.^1^ Similarly, high seroprevalence in South Africa did not prevent Omicron variant epidemics in 2022.^20^

Our study was subject to several important limitations. Firstly, those who participated may have differed with regards to unmeasured behavioural factors influencing seropositivity. Slightly less than 20% of participants were lost to follow-up at the last data collection, although efforts were made to follow-up by phone calls and home visits. Although the reasons for attrition were unknown, those not followed-up were moderately less likely to be vaccinated, suggesting possible association with other health-related behaviours not accounted for by our non-response adjustments. The combined effects of these selection biases on our estimates are difficult to predict.

Secondly, misclassification of infection status based on serology, and of case and vaccination status based on registry data and self-report, are additional, important potential sources of bias. In estimating prior-infection prevalence in the unvaccinated, our sensitivity analyses provided a range of point estimates considering the plausible combined effects of these biases. These remained within approximately ±15% of the primary analysis estimates. For the multivariable analyses of factors associated with recent infection we only presented results where the effects of misclassification based on serology were likely to be negligible. However, misclassification of case and vaccination status may have affected the results of both multivariable analyses. Given these analyses were also subject to possible confounding via sampling time and unmeasured factors, the results should be considered hypothesis-generating rather than confirmatory. Importantly, we were unable to distinguish immunity from vaccination and/or natural infection in vaccinated individuals. Our assessments of antibody kinetics after infection or vaccination may have been biased towards slower decay because we were unable to exclude individuals with undetected infection between measurements. Our estimates therefore represent a plausible lower limit on decay.

Thirdly, extrapolation of unvaccinated case ascertainment to the general population in our study may have been biased by plausible differences in symptoms or test-seeking behaviour, although the direction of this bias is difficult to predict. Care should be taken in comparison to estimates in other settings obtained with different approaches. Additionally, the discrepancies between questionnaire and vaccination data registry may be due to recall bias to report previous 3-month vaccination status.

With the systematic sampling for coverage across age and geography, our study allowed us to serologically track the nationwide progress of ongoing COVID-19 infection dynamics and vaccination delivery in Mongolia during 2020 and 2021. Excellent vaccine roll-out potentially played a pivotal role in limiting severe disease and mortality in Mongolia. This longitudinal seroepidemiological investigation confirms delayed introduction of SARS-CoV-2 through stringent public health measures until vaccinations became available. High seroprevalence in Mongolia by late 2021 did not prevent the Omicron epidemic in 2022. The role of paediatric SARS-CoV-2 vaccination remains unclear, due the seroprevalence rate and vaccine preventable COVID-19 disease in this age group. In future, follow-up of the same individuals and protective immunity levels should be monitored for the ongoing COVID-19 pandemic and its emerging escape variants.

## Contributors

BC served as principal investigator for the project. UM, OE, KN conducted planning and coordination. LL, TE, IB, has been supervisors. BB, ZD, OA, BM, AG, OB, GE, BS, EN, MD, UC did data collection and assisted laboratory work. AA, KE, UsM, BB, RE, TB, MyD, TA, ZN, KT, KT, EB, AB, OB, BayB, BatzB, GZ, GB assisted data collection and conducted laboratory work. BB, lead data entry and archiving. CB, NM, JM, DP developed the data analysis plan in consultation with other authors. CB conducted data analysis. RE, BC, UM, BB, CB, NM, JM, DP came up with first manuscript. Writing and conceptualisation has been conducted by RE, UM and BC. TE, LL, IB, ETs conducted manuscript editing and revisions. All authors read the final version of manuscript and contributed intellectually.

## Data sharing statement

Data sharing will be considered under negotiation with authors and IRB committee at the Ministry of Health, Mongolia, for deidentified serological testing results, age, sex, locational information can be made available. Requests should be delivered to the corresponding author. Compilation of this serosurvey is also made available to WHO Registry system.

## Declaration of interests

We declare no competing interests.

## Appendix A Supplementary data

Cyrillic Mongolian translation of abstract
