# Supplementary material for: SARS-CoV-2 seroepidemiology in Mongolia, 2020–2021: a longitudinal national study
Source: Lancet Reg Health West Pac. 2023 Apr 10;36:100760. doi: 10.1016/j.lanwpc.2023.100760 (PMC10084888; doi:10.1016/j.lanwpc.2023.100760)
Supplement: Cyrillic Mongolian translation of abstract [file mmc1.docx]

Supplementary material 1. Cyrillic Mongolian abstract

*This translation in Cyrillic Mongolian was submitted by the authors and we reproduce it as supplied. It has not been peer reviewed. Our editorial processes have only been applied to the original abstract in English, which should serve as reference for this manuscript.*

*Cyrillic Mongolian abstract*

**Монгол Улсын SARS-CoV-2 ийлдэс тархварзүй, 2020-2021: Үндэсний хэмжээний, урт хугацаанд дагах судалгаа**

**Үндэслэл**

Дэлхий дахиныг хамарсан КОВИД-19 цартахал хөгжиж буй орнуудад төдийлөн бүрэн судлагдаагүй байна. Бага-дунд орлоготой орны нэг Монгол улсын хувьд 2020 он эхлэх үеэс халдварын хяналтын хатуу арга хэмжээг авч хэрэгжүүлснээр 2021 оны 2 дугаар сард вакцин хэрэглээнд нэвтрэх хүртэл халдвар өргөн хүрээгээр тархахаас сэргийлж чадсан. Бид 2020-2021 онд Монгол улсын хүн амын дундах SARS-CoV-2-ийн ийлдэс тархварзүй, халдварт нөлөөлөх хүчин зүйлсийг тодорхойллоо.

**Аргазүй**

ДЭМБ-ын Юнити судалгааны стандарт протоколыг ашиглан урт хугацааны ийлдэс тархварзүйн судалгааг гүйцэтгэсэн. Судалгаанд нийт 5000 оролцогчийг хамруулан 2020 оны 10 сараас 2021 оны 12 сар хүртэл 4 удаагийн давтамжтай мэдээлэл, шинжилгээний материал цуглуулсан. Монгол орны хүн амаас насаар бүлэглэсэн, олон шатат кластер түүвэрлэлтийн аргаар оролцогчийг сонгон хамруулсан судалгааг эрүүл мэндийн анхан шатны байгууллагуудыг түшиглэн хийлээ. Судалгаанд хамрагдагсдын ийлдсэнд SARS-CoV-2-ийн эсрэг нийт эсрэгбиеийн илэрц, SARS-CoV-2-ийн эсрэг анти-спайк IgG-ийн болон саармагжуулагч эсрэгбиеийн титрийг тодорхойлсон. Түүнчлэн судалгааны энэхүү үр дүнг КОВИД-19 батлагдсан тохиолдол, нас баралтын мэдээлэл болон вакцинжуулалтын бүртгэлтэй уялдуулан судалсан. Хүн амын дундах ийлдэс тархварзүй, вакцинд хамрагдалт, болон халдварын тархалтыг тооцоолохын урьд вакцинд хамрагдаагүй хүн амыг тодорхойллоо.

**Үр дүн**

Судалгааны сүүлийн шат буюу 2021 оны төгсгөл хүртэл нийт оролцогчдын 82% (n=4088) судалгаанд бүрэн хамрагдсан. Монгол улс дахь SARS-CoV-2-ийн эсрэг нийт эсрэгбиеийн илэрц 2020 оны төгсгөлөөс 2021 оны төгсгөл хүртэл 1·5% (95%CI: 1·2-2·0)-аас 82·3% (95%CI: 79·5-84·8) хүртэл нэмэгдсэн байна. Судалгааны сүүлийн шатанд хүн амын 62·4% (95%CI: 60·2-64·5) вакцинжуулалтад хамрагдсан, харин вакцинд хамрагдаагүй хүн амын 64·5% (95%CI: 59·7-69·0) халдварт өртсөн байна. Вакцинд хамрагдаагүй хүн амын дундах хуримтлагдсан халдварын төвшин 22·8% (95%CI: 19·1%–26·9%), халдварт өртсөн хүмүүсийн дундах нас баралтын харьцаа 0·100% (95%CI: 0·088–0·124) тодорхойлогдсон. Судалгааны бүхий л шатанд эрүүл мэндийн ажилтанд КОВИД-19 халдвар батлагдах магадлал хамгийн өндөр байлаа. 2021 оны эхний хагасд эрэгтэйчүүд (1·72(95%CI: 1·33–2·22)) болон 20-с дээш настай насанд хүрэгчид (12·70(95%CI: 8·14–20·26)) ийн ийлдэс-урвалжих магадлал бусад бүлэгтэй харьцуулахад өндөр байв. 2021 оны төгсгөлд ийлдэс-эерэг хүн амын дунд саармагжуулагч эсрэгбиеийн илэрц 87·1% (95%CI: 82·3%-90·8%) тодорхойлогдсон.

**Дүгнэлт**

Монгол улсын хүн амын дунд SARS-CoV-2-ийн ийлдэс эерэг төвшин 2020 болон 2021 оны эхэн үед бага байснаа 2021 оны сүүлийн 3 сард халдварын нөхцөл байдал болон хүн амыг богино хугацаанд дархлаажуулсны үр дүнд эрчимтэй нэмэгдсэн байна. Хүн амын төвшний мэдээллийг нэмэлтээр ашигласан энэхүү судалгаагаар SARS-CoV-2-ийн ийлдэс хөдлөлзүйг үндэсний хэмжээнд тодорхойллоо. Судалгааны үр дүнгээр тогтоосон халдварын батлагдсан тохиолдол, нас баралт, дархлаажуулалтын төвшин албан ёсоор мэдээлсэн үр дүнтэй нийцэж байна. Хүн амын дундах ийлдэс эерэгийн төвшин 2021 оны эцсийн байдлаар өндөр тодорхойлогдсоны дараа SARS-CoV-2-ийн Омикрон хувилбарын халдвар дэгдэлт эрчимтэй явагдсан.

**Санхүүжилт**

Энэхүү судалгааг Дэлхийн эрүүл мэндийн байгууллага, ДЭМБ ЮНИТИ Судалгааны санаачилга, КОВИД-19-ийн хариу арга хэмжээний Solidarity сан, Холбооны Бүгд Найрамдах Германы Улсын Эрүүл мэндийн яамны дэргэдэх КОВИД-19-ийн судалгаа хөгжлийн төвийн санхүүжилтээр хийж гүйцэтгэв. Судалгааны зарим хэсгийг Монгол улсын ЗГ, Эрүүл мэндийн яам санхүүжүүлсэн.

**Түлхүүр үг**

КОВИД-19; SARS-CoV-2; Дархлаа тогтоц; Монгол улс; Тархварзүй; Цартахал; Ийлдэс тархварзүйн судалгаа; Ийлдэс тархалт
