# Supplementary material for: SARS-CoV-2 seroepidemiology in Mongolia, 2020–2021: a longitudinal national study
Source: Lancet Reg Health West Pac. 2023 Apr 10;36:100760. doi: 10.1016/j.lanwpc.2023.100760 (PMC10084888; doi:10.1016/j.lanwpc.2023.100760)
Supplement: Tables S1–S14 and Figures S1–S4 [file mmc2.docx]

# **Supplementary material 2**

| **Content** | **Page** |
| --- | --- |
| Supplementary results | 2 |
| **Table S1.** Wantai SARS-CoV-2 Ab ELISA assay sensitivity and specificity estimates from the literature.  **Table S2.** Symptoms used to define symptomatic COVID-19.  **Table S3.** Sample collection dates by survey round.  **Table S4.** Comparison of participants by response/nonresponse at subsequent survey rounds.  **Table S5.** Estimated SARS-CoV-2 seroprevalence in the Mongolian population at each survey round.  **Table S6.** Estimated proportion of the Mongolian population that had received at least one dose of COVID-19 vaccine at each survey round.  **Table S7.** Estimated prevalence of prior SARS-CoV-2 infection in the unvaccinated Mongolian population at each survey round.  **Table S8.** Characteristics of seropositive participants at the third and fourth rounds by availability of Wantai SARS-CoV-2 NAbs ELISA result.  **Table S9.** Estimated proportion of seropositive population with positive Wantai NAbs ELISA result at the third and fourth survey rounds.  **Table S10.** Estimated proportion of the seropositive population ascertained as confirmed cases by the final survey round.  **Table S11.** Estimated cumulative infections and infection-fatality risk at fourth round.  **Table S12.** Estimated proportion of newly seropositive unvaccinated population reporting COVID-19 compatible symptoms in the prior 3 months.  **Table S13.** Characteristics of participants assessed for antibody kinetics after vaccination.  **Table S14.** Characteristics of participants assessed for antibody kinetics after confirmed infection.  **Figure S1.** Estimated predictive value of Wantai assay for PCR-confirmed infection.  **Figure S2.** Sample estimates of population age, sex, and regional distribution.  **Figure S3.** Sensitivity analyses for the estimated prevalence of prior SARS-CoV-2 infection in the unvaccinated population.  **Figure S4.** Antibody kinetics after two doses of COVID-19 vaccine or confirmed infection. |  |
| Study Protocol | 21 |
|  |  |

**Table S1. Reported sensitivity (overall or >14 days post-symptom onset/PCR) and specificity of the Wantai SARS-CoV-2 Ab ELISA assay for SARS-CoV-2 infection.**

| Reference: | Sensitivity: | Specificity: |
| --- | --- | --- |
| [Manufacturer](https://www.fda.gov/media/140929/download) insert (1) | 99% (>14 days post-symptom onset) | 99% |
| Aubry *et al.* (2) | 90% (0-59 days post-positive PCR)  90% (60-119 days post-positive PCR)  95% (120-179 days post-positive PCR)  88% (≥180 days post-positive PCR) | - |
| Lerdsamran *et al.* (3) | 100% (overall) | 100% |
| Nyagwange *et al.* (4) | 95% (overall) | 98% |
| Nicholson *et al.* (5) | 100% (>14 days post-symptom onset) | 99.5% |
| Wiwe *et al.* (6) | 90.1% (overall) | 98.9% |
| Harritshoj *et al.* (7) | 96.7% (overall) | 99.5% |
| Bal *et al.*(8) | 100% (≥15 days post-symptom onset) | 100% |
| Bal *et al.* (9) | 100% (180–196 days post-symptom onset) | - |
| Lassaunière *et al.* (10) | 93% (overall) | 100% |
| Lou *et al.* (11) | 97.5% (overall) | 100% |
| Zhao *et al.* (12) | 93.1% (overall) | - |
| Chapuy-Regaud *et al.* (13) | 100% (overall) | 98% |

**Table S2. Symptoms used to define symptomatic COVID-19.**

| Fever |
| --- |
| Cough |
| Shortness of breath |
| Sore throat |
| Runny nose |
| Other respiratory symptoms |
| Chills |
| Vomiting |
| Nausea |
| Diarrhoea |
| Headache |
| Rash |
| Conjunctivitis |
| Muscle aches |
| Joint aches |
| Loss of appetite |
| Loss of smell |
| Loss of taste |
| Nose bleed |
| Fatigue |

**Table S3 Individual participant sample collection dates by survey round**

|  | **Sample collection date - median (range)** | | | |
| --- | --- | --- | --- | --- |
| **Region** | **First round** | **Second round** | **Third round** | **Fourth round** |
| Overall | 2020-10-21 (2020-10-13 - 2020-12-14) | 2021-02-23 (2021-01-18 - 2021-03-15) | 2021-06-03 (2021-05-24 - 2021-06-28) | 2021-09-13 (2021-09-03 - 2021-12-09) |
| Ulaanbaatar | 2020-11-27 (2020-10-14 - 2020-12-14) | 2021-02-28 (2021-01-20 - 2021-03-15) | 2021-06-13 (2021-06-04 - 2021-06-28) | 2021-11-15 (2021-10-28 - 2021-12-09) |
| Central region | 2020-10-16 (2020-10-13 - 2020-10-19) | 2021-01-23 (2021-01-18 - 2021-02-24) | 2021-05-29 (2021-05-25 - 2021-06-07) | 2021-09-06 (2021-09-03 - 2021-09-12) |
| Eastern region | 2020-10-14 (2020-10-13 - 2020-10-22) | 2021-01-26 (2021-01-18 - 2021-01-28) | 2021-05-28 (2021-05-24 - 2021-06-02) | 2021-09-07 (2021-09-06 - 2021-09-15) |
| Khangai region | 2020-10-17 (2020-10-13 - 2020-10-23) | 2021-01-23 (2021-01-18 - 2021-01-28) | 2021-06-01 (2021-05-25 - 2021-06-04) | 2021-09-09 (2021-09-03 - 2021-09-13) |
| Western region | 2020-10-30 (2020-10-16 - 2020-11-21) | 2021-02-24 (2021-01-20 - 2021-02-27) | 2021-05-28 (2021-05-25 - 2021-05-30) | 2021-09-06 (2021-09-05 - 2021-10-09) |

**Table S4. Comparison of followed-up and non-followed up participants by survey round**

|  | **Second round** | | | **Third round** | | | **Fourth round** | | |
| --- | --- | --- | --- | --- | --- | --- | --- | --- | --- |
|  | **Follow up (N=4524)** | **No follow up (N=476)** | **p-value** | **Follow up (N=4255)** | **No follow up (N=745)** | **p-value** | **Follow up (N=4088)** | **No follow up (N=912)** | **p-value** |
| **Sex** | | | | | | | | | |
| Female | 2958 (65.4%) | 284 (59.7%) | 0.0148 | 2799 (65.8%) | 443 (59.5%) | 0.001 | 2701 (66.1%) | 541 (59.3%) | <0.001 |
| Male | 1566 (34.6%) | 192 (40.3%) |  | 1456 (34.2%) | 302 (40.5%) |  | 1387 (33.9%) | 371 (40.7%) |  |
| **Age group (years)** | | | | | | | | | |
| 0-4 | 356 (7.9%) | 66 (13.9%) | <0.001 | 335 (7.9%) | 87 (11.7%) | <0.001 | 192 (4.7%) | 73 (8.0%) | <0.001 |
| 5-9 | 545 (12.0%) | 65 (13.7%) |  | 494 (11.6%) | 116 (15.6%) |  | 504 (12.3%) | 148 (16.2%) |  |
| 10-14 | 420 (9.3%) | 66 (13.9%) |  | 391 (9.2%) | 95 (12.8%) |  | 398 (9.7%) | 105 (11.5%) |  |
| 15-19 | 287 (6.3%) | 49 (10.3%) |  | 270 (6.3%) | 66 (8.9%) |  | 294 (7.2%) | 86 (9.4%) |  |
| 20-29 | 692 (15.3%) | 64 (13.4%) |  | 638 (15.0%) | 118 (15.8%) |  | 558 (13.6%) | 156 (17.1%) |  |
| 30-39 | 776 (17.2%) | 66 (13.9%) |  | 726 (17.1%) | 116 (15.6%) |  | 740 (18.1%) | 137 (15.0%) |  |
| 40-49 | 646 (14.3%) | 46 (9.7%) |  | 609 (14.3%) | 83 (11.1%) |  | 590 (14.4%) | 103 (11.3%) |  |
| 50-59 | 483 (10.7%) | 30 (6.3%) |  | 484 (11.4%) | 29 (3.9%) |  | 477 (11.7%) | 52 (5.7%) |  |
| 60-69 | 215 (4.8%) | 17 (3.6%) |  | 211 (5.0%) | 21 (2.8%) |  | 231 (5.7%) | 33 (3.6%) |  |
| 70+ | 104 (2.3%) | 7 (1.5%) |  | 97 (2.3%) | 14 (1.9%) |  | 104 (2.5%) | 19 (2.1%) |  |
| **Region** | | | | | | | | | |
| Ulaanbaatar | 2072 (45.8%) | 228 (47.9%) | <0.001 | 1905 (44.8%) | 395 (53.0%) | <0.001 | 1807 (44.2%) | 493 (54.1%) | <0.001 |
| Central region | 840 (18.6%) | 60 (12.6%) |  | 820 (19.3%) | 80 (10.7%) |  | 827 (20.2%) | 73 (8.0%) |  |
| Eastern region | 573 (12.7%) | 27 (5.7%) |  | 550 (12.9%) | 50 (6.7%) |  | 538 (13.2%) | 62 (6.8%) |  |
| Khangai region | 573 (12.7%) | 27 (5.7%) |  | 547 (12.9%) | 53 (7.1%) |  | 532 (13.0%) | 68 (7.5%) |  |
| Western region | 466 (10.3%) | 134 (28.2%) |  | 433 (10.2%) | 167 (22.4%) |  | 384 (9.4%) | 216 (23.7%) |  |
| **Ethnicity** | | | | | | | | | |
| Khalkh | 3732 (82.5%) | 341 (71.6%) | <0.001 | 3527 (82.9%) | 547 (73.4%) | <0.001 | 3395 (83.0%) | 678 (74.3%) | <0.001 |
| Kazakh | 215 (4.8%) | 100 (21.0%) |  | 181 (4.3%) | 134 (18.0%) |  | 159 (3.9%) | 156 (17.1%) |  |
| Buriad | 208 (4.6%) | 6 (1.3%) |  | 205 (4.8%) | 9 (1.2%) |  | 200 (4.9%) | 14 (1.5%) |  |
| Others | 204 (4.5%) | 22 (4.6%) |  | 188 (4.4%) | 38 (5.1%) |  | 186 (4.6%) | 40 (4.4%) |  |
| Missing | 165 (3.6%) | 7 (1.5%) |  | 154 (3.6%) | 17 (2.3%) |  | 148 (3.6%) | 24 (2.6%) |  |
| **Occupation** | | | | | | | | | |
| Healthcare worker | 569 (12.6%) | 19 (4.0%) | <0.001 | 510 (12.0%) | 78 (10.5%) | 0.261 | 471 (11.5%) | 117 (12.8%) | 0.293 |
| Other | 3955 (87.4%) | 457 (96.0%) |  | 3745 (88.0%) | 667 (89.5%) |  | 3617 (88.5%) | 795 (87.2%) |  |
| **Any medical comorbidity** | | | | | | | | | |
| No | 3480 (76.9%) | 215 (45.2%) | 0.104 | 3242 (76.2%) | 453 (60.8%) | <0.001 | 3103 (75.9%) | 592 (64.9%) | <0.001 |
| Yes | 1044 (23.1%) | 49 (10.3%) |  | 1006 (23.6%) | 87 (11.7%) |  | 982 (24.0%) | 111 (12.2%) |  |
| Missing | 0 (0%) | 212 (44.5%) |  | 7 (0.2%) | 205 (27.5%) |  | 3 (0.1%) | 209 (22.9%) |  |
| **Positive COVID-19 test in prior 3 months recorded in national registry*** | | | | | | | | | |
| No | 4507 (99.6%) | 472 (99.2%) | 0.924 | 4121 (96.9%) | 717 (96.2%) | 0.951 | 3494 (85.5%) | 802 (87.9%) | 0.0665 |
| Yes | 6 (0.1%) | 0 (0%) |  | 126 (3.0%) | 21 (2.8%) |  | 581 (14.2%) | 108 (11.8%) |  |
| Missing | 11 (0.2%) | 4 (0.8%) |  | 8 (0.2%) | 7 (0.9%) |  | 13 (0.3%) | 2 (0.2%) |  |
| **COVID-19 vaccination recorded in national registry*** | | | | | | | | | |
| No | 4400 (97.3%) | 470 (98.7%) | 0.00687 | 1929 (45.3%) | 450 (60.4%) | <0.001 | 1465 (35.8%) | 414 (45.4%) | <0.001 |
| Yes | 113 (2.5%) | 2 (0.4%) |  | 2318 (54.5%) | 288 (38.7%) |  | 2610 (63.8%) | 496 (54.4%) |  |
| Missing | 11 (0.2%) | 4 (0.8%) |  | 8 (0.2%) | 7 (0.9%) |  | 13 (0.3%) | 2 (0.2%) |  |

*To compare registry recorded vaccination and case status we assumed all non-followed-up participants would have been sampled on the median sampling date of their respective cluster. P-values from Chi-Squared test.

**Table S5. Estimated SARS-CoV-2 seroprevalence in the Mongolian population at each survey round**

| **Seroprevalence % (95% CI)** | | | | |
| --- | --- | --- | --- | --- |
|  | **First** | **Second** | **Third** | **Fourth** |
| Overall | 1.5 (1.2- 2.0) | 1.5 (1.1- 2.1) | 51.0 (47.6-54.4) | 80.6 (77.8-83.1) |
| Age group (years) | | | | |
| 0-4 | 1.3 (0.5- 3.2) | 0.3 (0.1- 1.5) | 10.9 (6.8-16.9) | 39.7 (28.8-51.8) |
| 5-9 | 0.7 (0.3- 1.5) | 1.0 (0.3- 3.4) | 12.6 (8.8-17.5) | 43.0 (34.2-52.3) |
| 10-14 | 1.9 (0.9- 3.7) | 2.1 (1.0- 4.4) | 13.9 (10.6-18.0) | 62.6 (56.1-68.8) |
| 15-19 | 1.5 (0.5- 4.1) | 1.8 (0.6- 5.6) | 44.9 (38.4-51.5) | 94.1 (88.8-96.9) |
| 20-29 | 1.9 (1.0- 3.6) | 1.2 (0.5- 2.8) | 73.2 (67.9-78.0) | 95.1 (90.6-97.5) |
| 30-39 | 2.3 (1.1- 4.7) | 2.8 (1.6- 4.8) | 73.9 (65.0-81.2) | 94.2 (91.0-96.3) |
| 40-49 | 1.6 (0.8- 3.4) | 1.6 (0.9- 2.7) | 74.9 (69.6-79.5) | 93.0 (88.8-95.7) |
| 50-59 | 1.3 (0.5- 3.7) | 0.9 (0.2- 3.7) | 71.8 (65.9-77.1) | 95.6 (92.7-97.3) |
| 60-69 | 0.8 (0.2- 3.0) | 1.8 (0.5- 6.7) | 60.9 (49.7-71.1) | 89.3 (79.4-94.7) |
| 70+ | 0.0 (0.0- 0.0) | 1.1 (0.2- 6.2) | 42.7 (31.9-54.2) | 85.4 (74.4-92.1) |
| Sex | | | | |
| Female | 1.8 (1.1- 2.8) | 2.0 (1.2- 3.3) | 55.9 (51.5-60.2) | 85.3 (82.4-87.8) |
| Male | 1.3 (0.7- 2.3) | 1.1 (0.6- 2.2) | 45.8 (42.1-49.6) | 75.6 (69.4-80.9) |
| Occupation | | | | |
| Healthcare worker | 1.8 (0.9- 3.6) | 1.8 (0.8- 3.9) | 79.9 (73.3-85.2) | 96.0 (92.5-97.9) |
| Other | 1.9 (1.1- 3.1) | 1.8 (1.2- 2.7) | 72.5 (67.3-77.2) | 94.1 (92.1-95.7) |
| Ethnicity | | | | |
| Khalkh | 1.6 (1.2- 2.1) | 1.6 (1.1- 2.2) | 51.0 (46.4-55.5) | 79.7 (76.8-82.4) |
| Kazakh | 1.8 (1.5- 2.2) | 2.7 (2.1- 3.4) | 43.1 (35.3-51.3) | 88.5 (81.8-93.0) |
| Buriad | 0.7 (0.3- 1.7) | 0.9 (0.3- 2.6) | 36.2 (28.7-44.5) | 75.0 (65.3-82.6) |
| Others | 0.8 (0.1- 6.2) | 0.9 (0.3- 3.1) | 56.9 (44.8-68.3) | 83.8 (76.2-89.3) |
| Region | | | | |
| Ulaanbaatar | 1.5 (0.9- 2.2) | 0.7 (0.4- 1.2) | 65.5 (62.1-68.8) | 92.2 (90.1-93.9) |
| Central region | 2.0 (1.4- 2.9) | 2.7 (1.9- 3.8) | 48.0 (39.1-57.0) | 71.1 (65.7-76.0) |
| Eastern region | 1.0 (0.5- 1.9) | 1.1 (0.5- 2.4) | 33.5 (29.1-38.3) | 72.2 (68.9-75.3) |
| Khangai region | 1.5 (1.1- 2.0) | 2.2 (1.4- 3.3) | 36.1 (25.9-47.8) | 72.2 (71.4-73.0) |
| Western region | 1.4 (0.9- 2.3) | 1.8 (0.8- 4.1) | 40.7 (37.8-43.6) | 78.0 (60.3-89.2) |
| Ulaanbaatar district | | | | |
| Bayangol | 0.1 (0.0- 1.0) | 0.7 (0.3- 1.4) | 74.5 (65.0-82.2) | 94.3 (85.6-97.9) |
| Bayanzurkh | 2.0 (0.9- 4.5) | 1.5 (0.7- 2.9) | 57.1 (55.1-59.1) | 91.5 (88.5-93.7) |
| Chingeltei | 0.7 (0.3- 2.1) | 0.4 (0.1- 1.4) | 75.7 (70.4-80.3) | 91.7 (83.8-95.9) |
| Khan-Uul | 1.5 (0.4- 4.8) | 0.8 (0.1- 6.8) | 65.0 (61.1-68.8) | 89.7 (84.8-93.2) |
| Songinokhairkhan | 1.6 (0.6- 4.4) | 0.3 (0.0- 1.3) | 62.4 (53.7-70.5) | 92.3 (88.5-94.9) |
| Sukhbaatar | 2.4 (1.4- 3.9) | 0.8 (0.2- 3.3) | 65.8 (52.6-76.9) | 94.1 (85.4-97.7) |

**Table S6. Estimated proportion of the Mongolian population that had received at least one dose of COVID-19 vaccine at each survey round**

|  | **Proportion vaccinated - % (95% CI)** | | | |
| --- | --- | --- | --- | --- |
|  | **First** | **Second** | **Third** | **Fourth** |
| Overall | 0.0 (0.0- 0.0) | 2.2 (1.2- 3.9) | 52.7 (50.7-54.7) | 61.8 (59.7-63.9) |
| Age group (years) | | | | |
| 0-4 | 0.0 (0.0- 0.0) | 0.0 (0.0- 0.0) | 2.4 (1.1- 5.5) | 3.6 (1.1-11.4) |
| 5-9 | 0.0 (0.0- 0.0) | 0.0 (0.0- 0.0) | 2.1 (0.7- 5.7) | 2.3 (0.8- 6.5) |
| 10-14 | 0.0 (0.0- 0.0) | 0.0 (0.0- 0.0) | 1.1 (0.3- 4.4) | 24.5 (19.0-31.0) |
| 15-19 | 0.0 (0.0- 0.0) | 0.0 (0.0- 0.0) | 36.0 (29.8-42.7) | 71.8 (62.2-79.8) |
| 20-29 | 0.0 (0.0- 0.0) | 5.1 (2.8- 9.2) | 78.6 (74.0-82.7) | 83.2 (79.2-86.5) |
| 30-39 | 0.0 (0.0- 0.0) | 2.6 (1.3- 5.2) | 78.8 (74.0-82.9) | 85.1 (80.9-88.5) |
| 40-49 | 0.0 (0.0- 0.0) | 2.7 (1.6- 4.7) | 81.8 (77.4-85.5) | 84.7 (80.2-88.3) |
| 50-59 | 0.0 (0.0- 0.0) | 4.2 (2.1- 8.1) | 82.6 (76.9-87.2) | 84.1 (78.2-88.6) |
| 60-69 | 0.0 (0.0- 0.0) | 5.2 (1.3-18.8) | 85.7 (78.2-91.0) | 87.5 (80.9-92.0) |
| 70+ | 0.0 (0.0- 0.0) | 0.0 (0.0- 0.0) | 75.5 (63.4-84.6) | 83.3 (73.7-89.8) |
| Sex | | | | |
| Female | 0.0 (0.0- 0.0) | 3.2 (1.9- 5.5) | 59.7 (56.9-62.4) | 69.7 (66.6-72.6) |
| Male | 0.0 (0.0- 0.0) | 1.1 (0.6- 2.3) | 45.2 (42.5-48.0) | 53.3 (50.2-56.4) |
| Occupation | | | | |
| Healthcare worker | 0.0 (0.0- 0.0) | 14.9 (8.4-25.0) | 79.3 (75.1-83.0) | 85.8 (81.7-89.1) |
| Other | 0.0 (0.0- 0.0) | 1.1 (0.5- 2.8) | 80.6 (76.9-83.7) | 84.2 (80.9-87.0) |
| Ethnicity | | | | |
| Khalkh | 0.0 (0.0- 0.0) | 1.5 (0.8- 2.8) | 51.4 (48.9-54.0) | 60.4 (57.6-63.0) |
| Kazakh | 0.0 (0.0- 0.0) | 1.9 (0.2-13.7) | 56.3 (42.0-69.7) | 69.8 (46.1-86.2) |
| Buriad | 0.0 (0.0- 0.0) | 0.6 (0.0- 9.9) | 56.2 (46.7-65.3) | 66.6 (59.7-72.9) |
| Others | 0.0 (0.0- 0.0) | 2.2 (0.8- 5.6) | 56.9 (48.7-64.8) | 64.0 (56.6-70.7) |
| Region | | | | |
| Ulaanbaatar | 0.0 (0.0- 0.0) | 5.2 (3.3- 8.1) | 55.3 (52.4-58.1) | 64.1 (60.8-67.3) |
| Central region | 0.0 (0.0- 0.0) | 0.0 (0.0- 0.0) | 50.8 (46.3-55.4) | 59.9 (56.1-63.5) |
| Eastern region | 0.0 (0.0- 0.0) | 0.0 (0.0- 0.0) | 50.0 (43.8-56.2) | 60.0 (51.9-67.6) |
| Khangai region | 0.0 (0.0- 0.0) | 0.0 (0.0- 0.0) | 47.5 (44.6-50.3) | 56.8 (54.1-59.4) |
| Western region | 0.0 (0.0- 0.0) | 0.0 (0.0- 0.0) | 57.0 (50.1-63.6) | 67.9 (58.6-75.9) |
| Ulaanbaatar district | | | | |
| Bayangol | 0.0 (0.0- 0.0) | 9.7 (5.2-17.6) | 54.7 (44.9-64.1) | 58.8 (47.5-69.2) |
| Bayanzurkh | 0.0 (0.0- 0.0) | 0.3 (0.0- 2.2) | 48.7 (43.6-53.8) | 60.4 (53.3-67.1) |
| Chingeltei | 0.0 (0.0- 0.0) | 4.6 (1.0-18.3) | 55.7 (52.1-59.2) | 59.6 (54.8-64.3) |
| Khan-Uul | 0.0 (0.0- 0.0) | 6.7 (5.4- 8.4) | 51.0 (48.5-53.5) | 63.0 (59.2-66.6) |
| Songinokhairkhan | 0.0 (0.0- 0.0) | 7.1 (2.5-18.1) | 59.5 (56.7-62.3) | 68.7 (66.3-71.0) |
| Sukhbaatar | 0.0 (0.0- 0.0) | 3.6 (1.8- 7.0) | 62.5 (51.6-72.3) | 75.5 (64.5-84.0) |

**Table S7. Estimated prevalence of prior SARS-CoV-2 infection in the unvaccinated Mongolian population at each survey round**

|  | **Prior-infection prevalence - % (95% CI)** | | | |
| --- | --- | --- | --- | --- |
|  | **First** | **Second** | **Third** | **Fourth** |
| Overall | 0.6 (0.2- 1.0) | 1.3 (0.8- 2.0) | 25.4 (22.4-28.6) | 61.0 (54.5-67.1) |
| Age group (years) | | | | |
| 0-4 | 0.3 (0.0- 2.2) | 1.3 (0.0- 4.4) | 11.0 (6.2-18.3) | 38.8 (27.7-51.1) |
| 5-9 | 0.0 (0.0- 0.5) | 0.0 (0.0- 2.4) | 12.2 (8.3-17.4) | 45.5 (37.1-54.1) |
| 10-14 | 0.9 (0.0- 2.8) | 2.0 (0.5- 5.0) | 14.5 (11.0-18.9) | 54.5 (46.8-62.0) |
| 15-19 | 0.5 (0.0- 3.1) | 1.5 (0.0- 5.7) | 33.4 (25.8-42.0) | 90.6 (78.5-96.5) |
| 20-29 | 0.9 (0.0- 2.7) | 1.2 (0.2- 2.8) | 52.6 (37.5-67.2) | 95.7 (89.7-98.6) |
| 30-39 | 1.4 (0.1- 3.8) | 3.1 (1.3- 6.2) | 46.8 (36.0-58.0) | 88.0 (81.6-92.5) |
| 40-49 | 0.7 (0.0- 2.5) | 1.4 (0.5- 3.0) | 63.6 (55.5-71.1) | 97.1 (89.4-99.7) |
| 50-59 | 0.3 (0.0- 2.8) | 0.0 (0.0- 2.9) | 61.4 (47.3-73.9) | 95.7 (83.5-99.6) |
| 60-69 | 0.0 (0.0- 2.0) | 1.3 (0.0- 6.4) | 65.4 (41.1-83.8) | 80.3 (55.4-93.4) |
| 70+ | 0.0 (0.0- 0.0) | 0.1 (0.0- 5.3) | 47.6 (17.5-79.4) | 79.2 (43.2-95.6) |
| Sex | | | | |
| Female | 0.8 (0.2- 1.9) | 1.8 (0.8- 3.4) | 26.3 (22.8-30.2) | 65.3 (60.6-69.7) |
| Male | 0.3 (0.0- 1.3) | 0.8 (0.0- 2.1) | 24.6 (20.4-29.4) | 58.0 (48.5-67.0) |
| Occupation | | | | |
| Healthcare worker | 0.8 (0.0- 2.6) | 2.1 (0.7- 4.5) | 57.9 (44.3-70.4) | 97.1 (87.8-99.9) |
| Other | 0.9 (0.1- 2.2) | 1.6 (0.8- 2.7) | 54.5 (47.6-61.2) | 93.1 (89.3-95.7) |
| Ethnicity | | | | |
| Khalkh | 0.6 (0.2- 1.1) | 1.4 (0.8- 2.2) | 26.0 (22.4-29.9) | 60.4 (53.2-67.2) |
| Kazakh | 0.8 (0.5- 1.2) | 2.1 (1.5- 2.9) | 22.5 (7.1-51.1) | 71.5 (0.0- 100.0) |
| Buriad | 0.0 (0.0- 0.7) | 0.1 (0.0- 1.8) | 16.3 (1.1-66.9) | 56.1 (0.0- 100.0) |
| Others | 0.0 (0.0- 5.4) | 0.5 (0.0- 4.7) | 23.4 (13.7-36.7) | 67.6 (48.4-82.5) |
| Region | | | | |
| Ulaanbaatar | 0.5 (0.0- 1.3) | 0.9 (0.3- 1.7) | 37.8 (33.7-42.1) | 83.5 (79.2-87.1) |
| Central region | 1.1 (0.4- 2.0) | 2.2 (1.3- 3.6) | 20.7 (15.0-27.7) | 42.2 (32.3-52.8) |
| Eastern region | 0.0 (0.0- 0.9) | 0.8 (0.1- 1.8) | 12.4 (7.3-19.9) | 50.4 (41.7-59.1) |
| Khangai region | 0.5 (0.1- 1.0) | 1.6 (0.4- 3.8) | 17.2 (13.7-21.3) | 49.5 (47.2-51.8) |
| Western region | 0.4 (0.0- 1.3) | 1.1 (0.0- 3.6) | 15.7 (8.2-27.6) | 52.3 (24.4-78.8) |
| Ulaanbaatar district | | | | |
| Bayangol | 0.0 (0.0- 0.0) | 0.0 (0.0- 1.1) | 50.8 (43.9-57.6) | 91.0 (78.8-96.8) |
| Bayanzurkh | 1.0 (0.0- 3.6) | 2.2 (0.9- 4.2) | 31.8 (25.3-39.0) | 82.8 (72.9-89.7) |
| Chingeltei | 0.0 (0.0- 1.1) | 0.0 (0.0- 0.9) | 53.1 (43.0-63.1) | 85.5 (71.0-93.8) |
| Khan-Uul | 0.5 (0.0- 3.9) | 0.7 (0.0- 5.2) | 40.5 (33.5-47.9) | 78.8 (70.1-85.6) |
| Songinokhairkhan | 0.6 (0.0- 3.5) | 0.7 (0.0- 4.1) | 31.2 (22.8-40.9) | 80.0 (69.8-87.5) |
| Sukhbaatar | 1.4 (0.4- 3.0) | 1.1 (0.5- 2.1) | 27.2 (10.8-52.7) | 81.1 (62.1-92.1) |

**Table S8. Characteristics of seropositive participants at the third and fourth rounds by availability of Wantai SARS-CoV-2 Neutralising Antibodies (NAbs) ELISA result.**

|  | **Third round** | | **Fourth round** | |
| --- | --- | --- | --- | --- |
|  | **NAb result available (N=2185)** | **Not available (N=21)** | **NAb result available (N=3333)** | **Not available (N=6)** |
| **Sex** |  |  |  |  |
| Female | 1506 (68.9%) | 14 (66.7%) | 2254 (67.6%) | 4 (66.7%) |
| Male | 679 (31.1%) | 7 (33.3%) | 1079 (32.4%) | 2 (33.3%) |
| **Age group (years)** |  |  |  |  |
| 0-4 | 38 (1.7%) | 0 (0%) | 76 (2.3%) | 1 (16.7%) |
| 5-9 | 55 (2.5%) | 0 (0%) | 223 (6.7%) | 0 (0%) |
| 10-14 | 52 (2.4%) | 0 (0%) | 253 (7.6%) | 1 (16.7%) |
| 15-19 | 114 (5.2%) | 0 (0%) | 280 (8.4%) | 1 (16.7%) |
| 20-29 | 475 (21.7%) | 4 (19.0%) | 527 (15.8%) | 0 (0%) |
| 30-39 | 511 (23.4%) | 3 (14.3%) | 686 (20.6%) | 0 (0%) |
| 40-49 | 451 (20.6%) | 2 (9.5%) | 549 (16.5%) | 0 (0%) |
| 50-59 | 329 (15.1%) | 7 (33.3%) | 448 (13.4%) | 2 (33.3%) |
| 60-69 | 120 (5.5%) | 4 (19.0%) | 203 (6.1%) | 0 (0%) |
| 70+ | 40 (1.8%) | 1 (4.8%) | 88 (2.6%) | 1 (16.7%) |
| **Region** |  |  |  |  |
| Ulaanbaatar | 1254 (57.4%) | 20 (95.2%) | 1657 (49.7%) | 4 (66.7%) |
| Central region | 351 (16.1%) | 0 (0%) | 590 (17.7%) | 0 (0%) |
| Eastern region | 205 (9.4%) | 0 (0%) | 401 (12.0%) | 0 (0%) |
| Khangai region | 203 (9.3%) | 0 (0%) | 392 (11.8%) | 0 (0%) |
| Western region | 172 (7.9%) | 1 (4.8%) | 293 (8.8%) | 2 (33.3%) |
| **Ethnicity** |  |  |  |  |
| Khalkh | 1805 (82.6%) | 17 (81.0%) | 2758 (82.7%) | 5 (83.3%) |
| Kazakh | 83 (3.8%) | 0 (0%) | 136 (4.1%) | 1 (16.7%) |
| Buriad | 86 (3.9%) | 0 (0%) | 154 (4.6%) | 0 (0%) |
| Others | 107 (4.9%) | 0 (0%) | 149 (4.5%) | 0 (0%) |
| Missing | 104 (4.8%) | 4 (19.0%) | 136 (4.1%) | 0 (0%) |
| **Occupation** |  |  |  |  |
| Healthcare worker | 321 (14.7%) | 7 (33.3%) | 409 (12.3%) | 1 (16.7%) |
| Other | 1864 (85.3%) | 14 (66.7%) | 2924 (87.7%) | 5 (83.3%) |
| **Any medical comorbidity** |  |  |  |  |
| No | 1563 (71.5%) | 12 (57.1%) | 2473 (74.2%) | 6 (100%) |
| Yes | 619 (28.3%) | 9 (42.9%) | 860 (25.8%) | 0 (0%) |
| Missing | 3 (0.1%) | 0 (0%) | 0 (0%) | 0 (0%) |

**Table S9. Estimated proportion of seropositive population with positive Wantai Neutralising Antibodies (NAbs) ELISA result at the third and fourth survey rounds.**

|  | **Percent NAb positive (95% CI)** | |
| --- | --- | --- |
|  | **Third** | **Fourth** |
| Overall | 61.7 (56.2-67.0) | 86.4 (81.7-90.1) |
| Age group (years) | | |
| 0-4 | 58.9 (30.1-82.6) | 87.8 (75.0-94.5) |
| 5-9 | 63.0 (48.1-75.8) | 90.2 (84.1-94.1) |
| 10-14 | 66.6 (49.2-80.4) | 91.8 (85.0-95.7) |
| 15-19 | 54.8 (41.2-67.6) | 89.9 (80.0-95.2) |
| 20-29 | 68.7 (62.4-74.4) | 86.3 (78.4-91.7) |
| 30-39 | 60.0 (53.4-66.2) | 87.5 (81.6-91.7) |
| 40-49 | 58.4 (49.2-67.1) | 82.9 (78.2-86.8) |
| 50-59 | 67.4 (58.3-75.3) | 84.4 (78.8-88.7) |
| 60-69 | 49.5 (36.7-62.4) | 79.7 (69.1-87.3) |
| 70+ | 49.0 (27.2-71.1) | 84.7 (69.6-93.0) |
| Sex | | |
| Female | 63.8 (57.9-69.3) | 86.5 (81.0-90.6) |
| Male | 59.1 (52.7-65.3) | 86.4 (81.6-90.1) |
| Occupation | | |
| Healthcare worker | 67.2 (58.8-74.6) | 86.9 (82.9-90.1) |
| Other | 62.6 (56.0-68.7) | 84.9 (79.5-89.1) |
| Ethnicity | | |
| Khalkh | 60.8 (54.3-67.0) | 85.6 (80.5-89.6) |
| Kazakh | 53.8 (34.7-71.9) | 88.9 (83.2-92.8) |
| Buriad | 39.7 (0.0- 100.0) | 81.5 (67.3-90.5) |
| Others | 67.2 (56.0-76.8) | 93.0 (85.3-96.8) |
| Region | | |
| Ulaanbaatar | 73.5 (68.5-78.0) | 95.3 (93.8-96.4) |
| Central region | 60.2 (44.3-74.1) | 78.7 (72.9-83.5) |
| Eastern region | 43.9 (38.9-49.0) | 76.7 (70.0-82.3) |
| Khangai region | 39.0 (31.5-47.0) | 80.6 (57.5-92.7) |
| Western region | 40.4 (28.6-53.5) | 78.2 (57.0-90.6) |
| Ulaanbaatar district | | |
| Bayangol | 83.1 (63.1-93.4) | 96.5 (91.6-98.6) |
| Bayanzurkh | 36.6 (30.2-43.6) | 93.4 (91.1-95.2) |
| Chingeltei | 79.0 (73.0-84.0) | 93.5 (90.8-95.4) |
| Khan-Uul | 66.7 (53.6-77.7) | 96.8 (87.3-99.3) |
| Songinokhairkhan | 91.3 (88.7-93.3) | 95.6 (91.9-97.7) |
| Sukhbaatar | 83.2 (77.4-87.7) | 96.0 (91.2-98.2) |

**Table S10. Estimated proportion of the unvaccinated ever-seropositive population ascertained as confirmed cases at the end of the fourth survey round.**

|  | **Case ascertainment % (95% CI)** |
| --- | --- |
| Overall | 22.0 (18.7-25.7) |
| Age group (years) | |
| 0-4 | 22.0 (13.0-34.8) |
| 5-9 | 18.6 (12.4-26.9) |
| 10-14 | 18.5 (12.3-26.9) |
| 15-19 | 12.4 (5.5-25.8) |
| 20-29 | 26.3 (19.1-35.1) |
| 30-39 | 28.2 (18.5-40.5) |
| 40-49 | 30.3 (20.6-42.2) |
| 50-59 | 23.2 (14.9-34.3) |
| 60-69 | 43.0 (20.4-69.0) |
| 70+ | 28.3 (8.5-62.4) |
| Region | |
| Ulaanbaatar | 29.9 (25.4-34.8) |
| Central region | 7.0 (2.9-15.5) |
| Eastern region | 29.2 (20.3-40.0) |
| Khangai region | 9.0 (3.4-21.8) |
| Western region | 22.1 (10.2-41.5) |

**Table S11. Estimated cumulative infections and infection-fatality risk at the end of fourth survey round.**

|  | **Total infected - n (95% CI)** | **Infection-fatality ratio - % (95% CI)** |
| --- | --- | --- |
| Overall | 1456109 (1245852-1714119) | 0.1010 (0.0858-0.1180) |
| Age group (years) | | |
| 0-4 | 80269 (50729-136211) | 0.0087 (0.0051-0.0138) |
| 5-9 | 123157 (84970-184819) | 0.0008 (0.0005-0.0012) |
| 10-14 | 107847 (74362-161956) | 0.0028 (0.0019-0.0040) |
| 15-19 | 126345 (60837-286855) | 0.0008 (0.0003-0.0016) |
| 20-29 | 220710 (165447-304425) | 0.0054 (0.0039-0.0073) |
| 30-39 | 275122 (191617-419758) | 0.0185 (0.0121-0.0266) |
| 40-49 | 156456 (112481-230169) | 0.0505 (0.0343-0.0702) |
| 50-59 | 140426 (95187-218386) | 0.1446 (0.0930-0.2133) |
| 60-69 | 41668 (25965-88000) | 0.9048 (0.4284-1.4519) |
| 70+ | 36270 (16412-120118) | 2.0292 (0.6127-4.4845) |

**Table S12. Estimated proportion of newly seropositive unvaccinated population reporting COVID-19 compatible symptoms in the prior 3 months.**

|  | **Symptomatic fraction - % (95% CI)** | | | |
| --- | --- | --- | --- | --- |
|  | **Third round** | | **Fourth round** | |
|  | **One symptom** | **Two symptoms** | **One symptom** | **Two symptoms** |
| Overall | 13.7 (8.7-20.8) | 10.1 (6.1-16.4) | 24.8 (19.4-31.1) | 18.6 (15.1-22.8) |
| Age group (years) | | | | |
| 0-4 | 22.5 (7.1-52.6) | 11.5 (3.2-33.6) | 11.9 (3.9-31.1) | 11.9 (3.9-31.1) |
| 5-9 | 6.7 (1.7-23.1) | 5.8 (1.2-24.1) | 18.5 (13.2-25.3) | 13.5 (8.9-19.8) |
| 10-14 | 9.0 (3.2-23.1) | 9.0 (3.2-23.1) | 27.5 (19.4-37.4) | 20.7 (13.4-30.5) |
| 15-19 | 9.8 (2.6-30.3) | 9.8 (2.6-30.3) | 12.4 (5.0-27.3) | 8.6 (2.8-23.8) |
| 20-29 | 22.0 (13.5-33.8) | 16.9 (9.0-29.5) | 40.5 (20.7-64.0) | 32.8 (14.1-59.3) |
| 30-39 | 9.8 (4.5-19.9) | 7.8 (3.5-16.5) | 41.1 (19.9-66.2) | 31.2 (10.5-63.7) |
| 40-49 | 15.1 (6.2-32.4) | 11.1 (3.5-30.0) | 49.2 (24.5-74.4) | 36.1 (20.7-55.1) |
| 50-59 | 23.8 (10.9-44.3) | 13.5 (4.3-35.4) | 36.5 (14.6-66.0) | 24.4 (12.4-42.4) |
| 60-69 | 9.2 (1.7-37.4) | 4.2 (0.4-32.6) | 46.9 (4.1-94.8) | 23.0 (1.3-87.2) |
| 70+ | 2.2 (0.0-80.8) | 2.2 (0.0-80.8) | 37.7 (2.8-92.7) | 37.7 (2.8-92.7) |

**Table S13. Characteristics of participants assessed for antibody kinetics after vaccination. Participants were assessed independently for anti-Spike IgG or neutralising antibody (NAb) kinetics if they had received two doses of vaccine and had at least two subsequent results available without interim vaccination or confirmed infection, with the first result obtained at least 28 days after the second dose of vaccine.**

|  | **anti-Spike IgG after vaccination (N=40)** | **NAbs after vaccination (N=183)** |
| --- | --- | --- |
| **Sex** |  |  |
| Female | 26 (65.0%) | 130 (71.0%) |
| Male | 14 (35.0%) | 53 (29.0%) |
| **Age (years)** |  |  |
| Median (range) | 37 (17, 79) | 37 (17, 79) |
| **Any medical comorbidity** |  |  |
| No | 27 (67.5%) | 132 (72.1%) |
| Yes | 13 (32.5%) | 51 (27.9%) |
| **1st vaccine type** |  |  |
| Comirnarty (Pfizer/BioNTech) | 0 (0%) | 4 (2.2%) |
| Vaxzevria (Oxford/AstraZeneca) | 8 (20.0%) | 39 (21.3%) |
| Vero Cell (Sinopharm) | 32 (80.0%) | 140 (76.5%) |
| **2nd vaccine type** |  |  |
| Comirnarty (Pfizer/BioNTech) | 0 (0%) | 4 (2.2%) |
| Vaxzevria (Oxford/AstraZeneca) | 8 (20.0%) | 39 (21.3%) |
| Vero Cell (Sinopharm) | 32 (80.0%) | 140 (76.5%) |
| **Dose interval (days)** |  |  |
| Median (range) | 27 (21, 56) | 28 (8, 56) |

**Table S14. Characteristics of participants assessed for antibody kinetics after confirmed infection Participants with confirmed infection were assessed independently for anti-Spike IgG or neutralising antibody (NAb) kinetics if they had at least two subsequent results available without interim vaccination, with the first result obtained at least 28 days post positive test.**

|  | **anti-Spike IgG after infection (N=10)** | **NAbs after infection (N=37)** |
| --- | --- | --- |
| **Sex** |  |  |
| Female | 7 (70.0%) | 26 (70.3%) |
| Male | 3 (30.0%) | 11 (29.7%) |
| **Age (years)** |  |  |
| Median (range) | 33 (20, 60) | 41 (20, 78) |
| **Any medical comorbidity** |  |  |
| No | 6 (60.0%) | 22 (59.5%) |
| Yes | 4 (40.0%) | 15 (40.5%) |


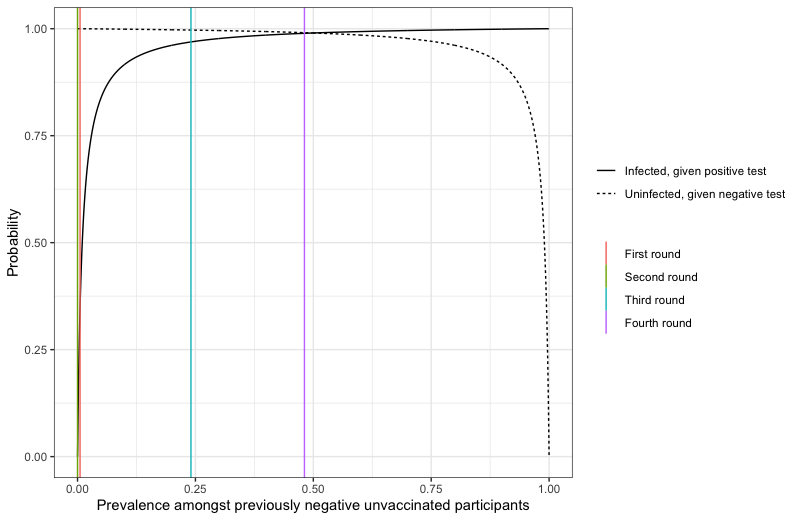


**Figure S1. Positive and negative predictive values of the Wantai SARS-CoV-2 total antibody assay, assuming 99% sensitivity and specificity. Prevalence estimates for infection (i.e. seropositivity adjusted for test performance) among previously seronegative participants for each round are shown as vertical lines.**


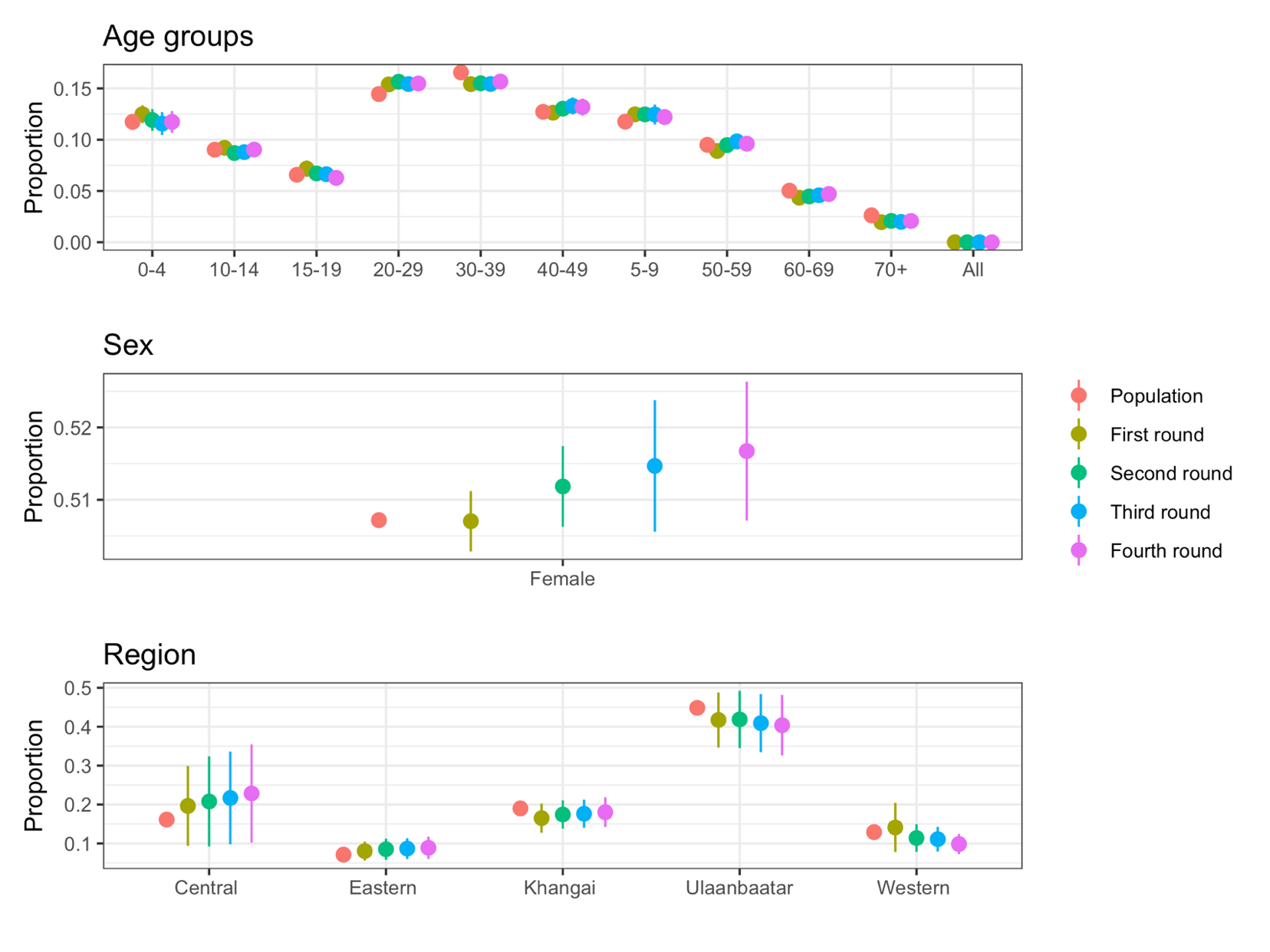


**Figure S1. Comparison of known population proportions for age group, sex, and region with corresponding sample estimates. Error bars show 95% confidence intervals.**


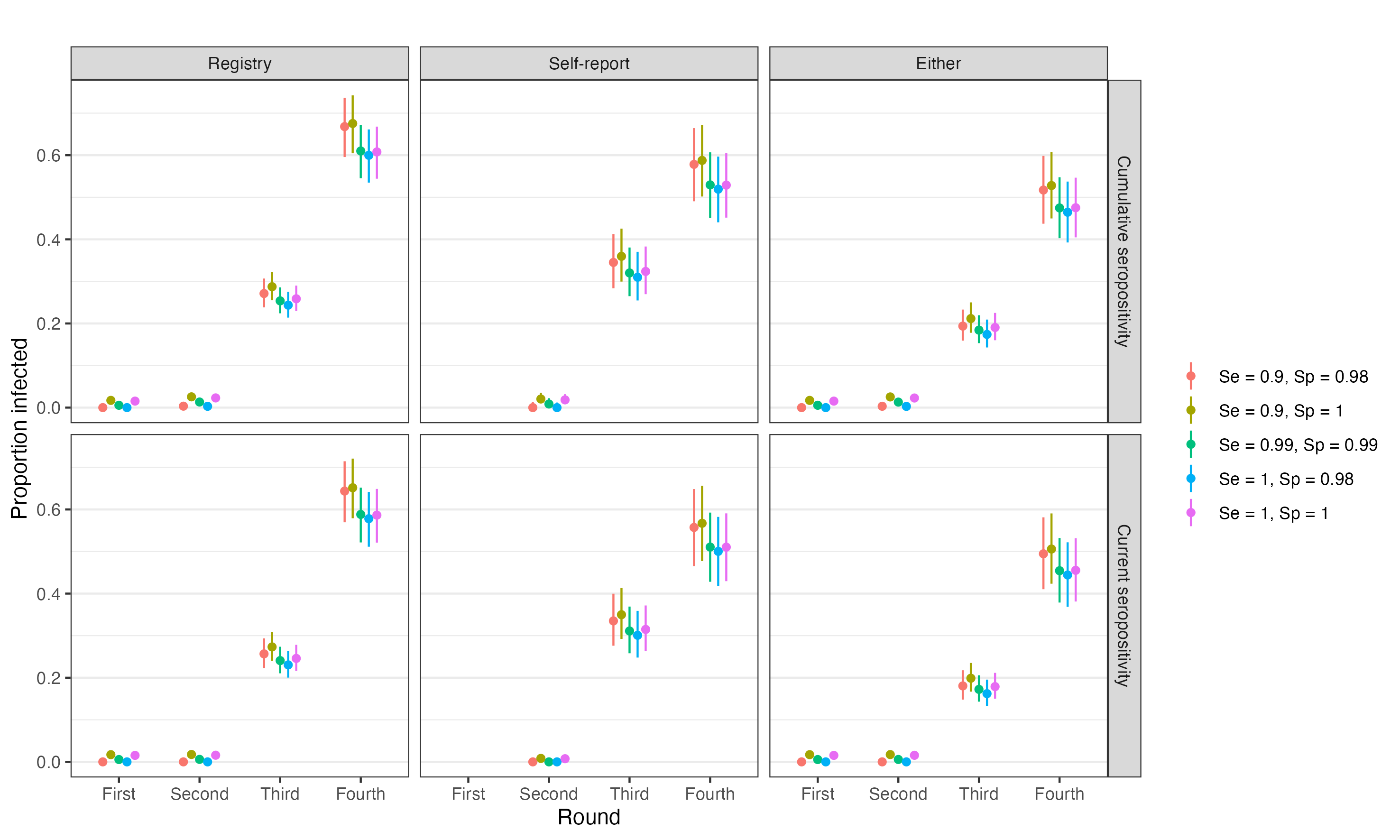


**Figure S2. Sensitivity analyses for prevalence of prior infection in the unvaccinated population by: i) ascertainment of vaccination status (registry-recorded, self-reported, or either; columns) ii) serological status used to define infection (seropositive at current round, or ever-seropositive; rows) iii) test performance (colour).**


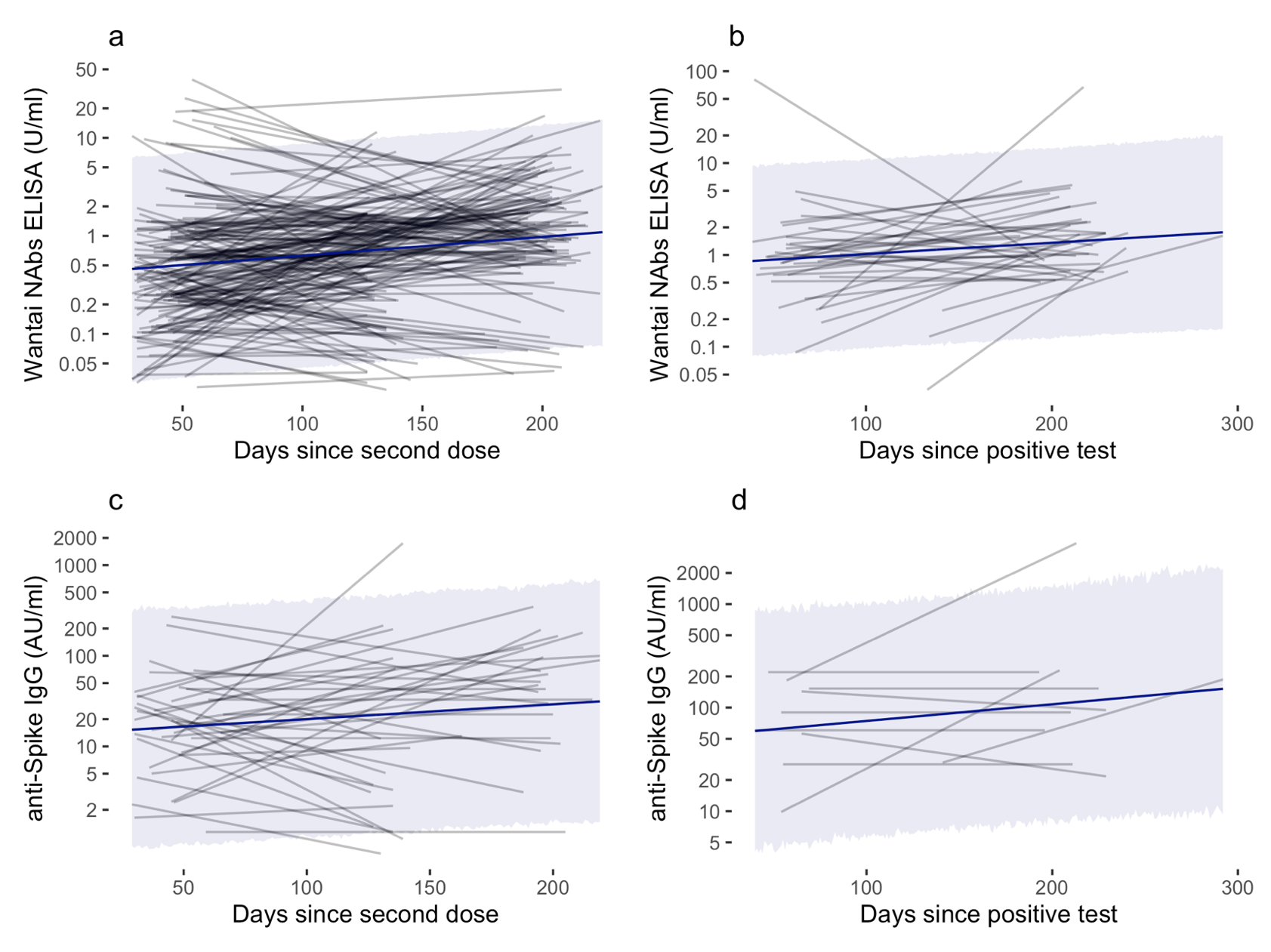


**Figure S4. Antibody kinetics after two doses of COVID-19 vaccine (a and c) or confirmed infection (b and d). Blue lines and ribbons show fixed effects prediction and median of individual 95% prediction intervals from linear random-intercept models.**

**References:**

1. Beijing Wantai Biological Pharmacy Enterprise Co. L. WANTAI SARS-CoV-2 Ab ELISA package insert. 2020.

2. Aubry A, Demey B, François C, Duverlie G, Castelain S, Helle F, et al. Longitudinal Analysis and Comparison of Six Serological Assays up to Eight Months Post-COVID-19 Diagnosis. J Clin Med. 2021;10(9).

3. Lerdsamran H, Mungaomklang A, Iamsirithaworn S, Prasertsopon J, Prasert K, Intalapaporn P, et al. Evaluation of different platforms for the detection of anti-SARS coronavirus-2 antibodies, Thailand. BMC Infectious Diseases. 2021;21(1):1213.

4. Nyagwange J, Kutima B, Mwai K, Karanja HK, Gitonga JN, Mugo D, et al. Comparative performance of WANTAI ELISA for total immunoglobulin to receptor binding protein and an ELISA for IgG to spike protein in detecting SARS-CoV-2 antibodies in Kenyan populations. Journal of Clinical Virology. 2022;146:105061.

5. Nicholson S, Karapanagiotidis T, Khvorov A, Douros C, Mordant F, Bond K, et al. Evaluation of 6 Commercial SARS-CoV-2 Serology Assays Detecting Different Antibodies for Clinical Testing and Serosurveillance. Open Forum Infectious Diseases. 2021;8(7):ofab239.

6. Wiwe EF, Carlsson ER, Rasmussen CL, Rasmussen P, Ougaard R, Hansen SI, et al. Long-Term Comparison of 7 SARS-CoV-2 Antibody Assays in the North Zealand Covid-19 Cohort. The Journal of Applied Laboratory Medicine. 2022:jfab173.

7. Harritshøj LH, Gybel-Brask M, Afzal S, Kamstrup PR, Jørgensen CS, Thomsen MK, et al. Comparison of 16 Serological SARS-CoV-2 Immunoassays in 16 Clinical Laboratories. Journal of Clinical Microbiology. 2021;59(5):e02596-20.

8. Bal A, Pozzetto B, Trabaud MA, Escuret V, Rabilloud M, Langlois-Jacques C, et al. Evaluation of High-Throughput SARS-CoV-2 Serological Assays in a Longitudinal Cohort of Patients with Mild COVID-19: Clinical Sensitivity, Specificity, and Association with Virus Neutralization Test. Clin Chem. 2021;67(5):742-52.

9. Bal A, Trabaud M-A, Fassier J-B, Rabilloud M, Saker K, Langlois-Jacques C, et al. Six-month antibody response to SARS-CoV-2 in healthcare workers assessed by virus neutralization and commercial assays. Clin Microbiol Infect. 2021;27(6):933-5.

10. Lassaunière R, Frische A, Harboe ZB, Nielsen ACY, Fomsgaard A, Krogfelt KA, et al. Evaluation of nine commercial SARS-CoV-2 immunoassays. medRxiv. 2020:2020.04.09.20056325.

11. Lou B, Li T-D, Zheng S-F, Su Y-Y, Li Z-Y, Liu W, et al. Serology characteristics of SARS-CoV-2 infection after exposure and post-symptom onset. European Respiratory Journal. 2020;56(2):2000763.

12. Zhao J, Yuan Q, Wang H, Liu W, Liao X, Su Y, et al. Antibody Responses to SARS-CoV-2 in Patients With Novel Coronavirus Disease 2019. Clin Infect Dis. 2020;71(16):2027-34.

13. Chapuy-Regaud S, Miédougé M, Abravanel F, Da Silva I, Porcheron M, Fillaux J, et al. Evaluation of Three Quantitative Anti-SARS-CoV-2 Antibody Immunoassays. Microbiol Spectr. 2021;9(3):e0137621-e.

**Study Protocol**


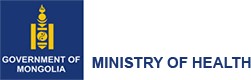

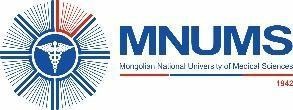

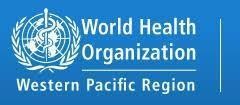


## Population-based age-stratified sero-epidemiological investigation protocol for COVID-19 infection

**Novel Coronavirus (COVID-19) Population-Based Age-Stratified Sero-Epidemiological Study in Mongolia (A Prospective, Nationwide Study)**

1. **Project Title:** Novel Coronavirus (COVID-19) population-based age-stratified sero-epidemiological study in Mongolia (A Prospective, Nationwide Study)
2. **Sub Area:** Population-based age-stratified sero-epidemiological investigation protocol for COVID-19 infection
3. **Duration in months:** 12 months
4. **Project Category:** Applied Research Any other: NA

### Participating organizations:

Ministry of Health Mongolia

Mongolian National University of Medical Sciences The World Health Organization

### Principal Investigator:

Assoc.Prof Battogtokh Chimeddorj, MD, PhD

Head of Department of Microbiology and Infection Prevention Control, School of Biomedicine, Mongolian National University of Mongolia

### Advisors:

Academician Nyamadawa P, MD, ScD, General Virologist, Professional Committee for Virology, Ministry of Health, Mongolia and Advisor for the National Influenza Center

Professor Davaalkham D, MD, PhD, Dean School of Public Health, MNUMS Dr Linh-Vi LE, PhD, MSc, MA, Epidemiologist, WPRO, WHO

### Co-Investigators:

Professor Khurelbaatar N, MD, PhD, President, Mongolian National University of Medical Sciences (MNUMS)

Damdindorj B, MD, PhD, Vice President for Research and International relation, Mongolian National University of Medical Sciences

Darambazar G, MD, PhD, Dean, School of Biomedicine, MNUMS

Dr Oyunsuren E, MD, PhD, Senior Expert, Ministry of Health, Mongolia

Assoc Professor Tuvshinjargal Ts, MD, PhD, Dean, Mongolian National University of Medical Sciences

Dr Undram, MD, PhD, Senior Lecturer, Department of Family Medicine, School of Medicine, Mongolian National University of Medical Sciences

Khongorzul T, MD, PhD, Head, Department of Immunology, School of Biomedicine, MNUMS

Ariunzaya B, MD, PhD, Department of Immunology, School of Biomedicine, MNUMS

Zolmunkh N, MD, PhD, Department of Immunology, School of Biomedicine, MNUMS

Enkh-Amar B, MD, Department of Immunology, School of Biomedicine, MNUMS

Otgonjargal B, PhD, Department of Microbiology and Infection Prevention Control, School of Biomedicine, MNUMS

Battur M, MSc, Department of Microbiology and Infection Prevention Control, School of Biomedicine, MNUMS

Oyunbaatar A, MSc, Department of Microbiology and Infection Prevention Control, School of Biomedicine, MNUMS

Zolzaya D, MD, MSc, Department of Microbiology and Infection Prevention Control, School of Biomedicine, MNUMS

Anuujin G, MD, MSc, Department of Microbiology and Infection Prevention Control, School of Biomedicine, MNUMS

Batzaya B, MSc, Department of Laboratory, Microbiology section, Mongolia- Japan University Hospital, MNUMS

Gereltsetseg Z, Department of Department of Laboratory, Immunology section, Mongolia-Japan University Hospital, MNUMS

Batzorig B, PhD, Department of Epidemiology and Biostatistics, School of Public Health

Oyungerel D, MSc in Public Health, Department of Epidemiology and Biostatistics, School of Public Health

Mandukhai G, MSc in Public Health, Department of Epidemiology and Biostatistics, School of Public Health

Erkebulan M, MSc, Department of Epidemiology and Biostatistics, School of Public Health

Khangai E, MSc, Department of Family Medicine, School of Medicine Batkhuu B, MSc, Department of Family Medicine, School of Medicine

Tuyajargal.B, MSc, Department of Family Medicine, School of Medicine

Usukhbayar. M, MSc, Department of Family Medicine, School of Medicine

Assoc.Prof Myagmartseren D, MD, PhD, Department of Family Medicine, School of Medicine

Dr Roger Evans, Laboratory virologist, WPRO, WHO Dr Ariuntuya, MD, TO, WHE, WHO Mongolia CO

Summary

| **Novel Coronavirus (COVID-19) population-based age-stratified sero- epidemiological study in Mongolia (A Prospective, Nationwide Study)** | |
| --- | --- |
| **Study population** | Age-stratified random sample from general population |
| **Potential output and analysis** | Estimate or inform estimates of:   - Seroprevalence of antibodies to COVID-19 - Cumulative incidence of infection - Infection attack rates - Fraction of asymptomatic infection - Case fatality ratio |
| **Study design** | Prospective population-based convenience sample from the general population, stratified by age   - Longitudinal cohort investigation with serial sampling of the same individuals |
| **Study duration** | Serial sampling as a prospective cohort study  Total duration: **1 year** |
| **Minimum information and specimens to be obtained from**  **participants** | Data collection: Epidemiological data including basic demographics and clinical symptoms  Specimens: Serum samples to inform sero-  epidemiological inferences |

1. **Background**

Mongolia’s surveillance system for COVID-19 currently consists of reporting of case detection. As of 31 August 2020, there were 301 cases of COVID-19 detected and no deaths in Mongolia. Despite the low number of cases, there is potential for increases in cases when public health and social measures are relaxed and given that Mongolia shares a border with China, where the SARS-CoV-2 virus was first detected in Wuhan city in December 2019. In support of the Ministry of Health’s decision to increase understanding on the extent and characteristics of SARS-CoV-2 infection in the population of Mongolia, the National University of Mongolia and partners propose to adapt the UNITY early sero-epidemiological investigation protocols^1^.

As is the case with SARS-CoV-2, the detection and spread of an emerging respiratory pathogen are accompanied by uncertainty over the key epidemiological and serologic characteristics of the novel pathogen and particularly its transmissibility (i.e. ability to spread in a population) and its virulence (i.e. case-severity). To date initial surveillance has focused primarily on patients with symptoms or severe disease, and, as such, the full spectrum of the disease, including the extent and fraction of mild or asymptomatic infections that do not require medical attention are not clear. Estimates of the case fatality ratio, and other epidemiological parameters, will likely be lower than current estimates once the full spectrum of disease is able to be included in the denominator. In addition, the role of pre- symptomatic, asymptomatic or subclinical infections in human-to-human transmission of SARS-CoV-2 virus is not well understood.

With a novel coronavirus, initial seroprevalence in the population is assumed to be negligible due to the virus being novel in origin. Therefore, surveillance of antibody seropositivity in a population can allow inferences to be made about the extent of infection and about the cumulative incidence of infection in the population.

The proposed study will investigate the extent of infection, as determined by sero- positivity in the general population nationwide in Mongolia. We have tailored some aspects of the UNITY protocol to align with public health, laboratory and clinical systems

^1^ [https://www.who.int/emergencies/diseases/novel-coronavirus-2019/technical-guidance/early-investig](https://www.who.int/emergencies/diseases/novel-coronavirus-2019/technical-guidance/early-investigations)

in Mongolia and according to capacity, availability of resources and cultural appropriateness. By applying WHO’s standardized protocol and using testing kits provided by WHO, our data would be comparable to other countries. We will contribute aggregated data on seroprevalence and epidemiological exposures to WHO UNITY studies for regional and global analyses across many different settings for timely estimates of COVID-19 virus infection, severity and attack rates, as well as to inform public health responses and policy decisions. This is particularly important in the context of a novel respiratory pathogen, such as COVID-19.

1. **Study objectives**

The objectives for this sero-epidemiological investigation are as follows:

- 1. To measure the seroprevalence of antibodies to COVID-19 in the general population at urban, rural and tribal areas by sex and age groups across Mongolia.
  2. To estimate the fraction of asymptomatic, pre-symptomatic or subclinical infections in the population and by sex and age group.
  3. To determine risk factors for infection by comparing the exposures of infected and non- infected individuals.
  4. To contribute to determine the case fatality ratio.
  5. To contribute to an improved understanding of antibody kinetics following COVID-19 infection.

Note: Little is currently known about COVID-19 virus antibody kinetics. Asymptomatic infected persons may clear the virus more quickly than do symptomatic patients. Antibody titers in the asymptomatic persons are likely to be lower, if they seroconvert at all, than in infected patients exhibiting symptoms. These are considerations for the interpretation of any COVID-19 virus sero-epidemiological investigation. We will be taking this aspect into consideration.

1. **Methods**

### Study design

The sero-epidemiological investigation for COVID-19 virus infection is a **population- based, age- stratified prospective study**. It is intended to provide key epidemiological and serologic characteristics of SARS-CoV-2 virus in Mongolia.

Through UNITY, the WHO aims to gather data from countries around world; we will be contributing from Mongolia data from longitudinal follow-up of a cohort of randomly selected participants to increase understanding of various unknown facts about antibody kinetics in symptomatic and asymptomatic, incidence, attack rates, case fatality, and determinants of infection. The detection of seroprevalence over time in Mongolia will help inform the extent of infection in the community and the level of individuals susceptible to SARS-CoV-2 over time.

### We will conduct a longitudinal cohort investigation with serial sampling of the same individuals over four periods of time (every quarter).

We will take the first sample for sero-positivity after collecting all baseline information on Day 1.

Follow-up samples from the same individuals will be repeated at months 3, 6 and 9.

Repeated samples at 3, 6 and 9 months would help us to calculate cumulative incidence of sero-conversion in population at various sites in different age groups.

The follow-up of positives will enable us to understand antibody kinetics following COVID-19 infection.

### Sampling strategy and selection of clusters

In order to collect baseline data in the shortest possible time, we will be focusing on getting primary data within 2 months.

### Sampling method

Three stage sampling (major cities, provincial centers and soum) was performed to cover all socio and geographical regions of Mongolia. (Figure 1)

In the next stage, multistage randomized sampling was performed to select study participants. Overall, 5000 participants were divided into 50 clusters, and were calculated as 100 participants to be selected from each cluster. Based on the population density, a total of 27 clusters selected from aimags and 23 clusters from Ulaanbaatar city. At aimag level, one cluster selected from provincial center and 2 clusters from soums in each aimag.

For each cluster, potential participants will be selected from rosters at health centers, which consist of all residents in the cluster area. To include all age groups in the community, we will select study participants from local residents by the age stratified random sampling method.

**Fig.1 Multi-stage random sampling scheme**

|  | | Mongolian population | | | | 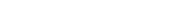 | | |
| --- | --- | --- | --- | --- | --- | --- | --- | --- |
|  | | | |  | | | | |
|  | |  | | | |  | | |
|  | Cities | | 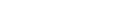 | | Rural areas | |  | |
|  | |  | | | |  | | |
|  |  | | |  | | | |  |
| District | |  | Province center | |  | | Soum | |
|  |  | | |  | | | |  |
| Khoroo | |  | Khoroo | |  | | Bag | |
|  |  | | |  | | | |  |
| Population | |  | Population | |  | | Population | |
|  |  | | |  | | | |  |
| Study population | |  | Study population | |  | | Study population | |

**Sample size**

The sample size is calculated for an expected 1% prevalence of SARS-CoV-2 antibodies with a 0.4% margin of error and design effect of 2 (see below sample size calculation). Although the sample size is calculated using a prevalence of 1%, given the current low case reports, we expect a prevalence of <1%. The target sample size would enable detection of seroconversion to obtain incidence. Table 1 shows study clusters from each region and capital city.

*Sample size calculation*

𝐷𝐸𝐹𝐹 ∗ 𝑁 ∗ 𝑍^2^ ∗ 𝑝 ∗ (1 − 𝑝) 2 ∗ 3238479 ∗ 1.96^2^ ∗ 1.0 ∗ 99.0

𝑛 = (𝑁 − 1) ∗ 𝑒^2^ + 𝑍^2^ ∗ 𝑝 ∗ (1 − 𝑝)

= 3238478

∗ 0.4^2^ + 1.96^2^ ∗ 1 ∗ 99 = 4750

- DEFF=2.0 /Design effect /
- N = 3 238 479 /Number of General Population of Mongolia, reported by National Statistical Organization, 2018/
- Z = 1.96 /critical value of standard normal distribution/
- P = 1% /expected sero-prevalence of antibodies to SARS-CoV-2,

o *Havers FP, Reed C, Lim T, et al. Seroprevalence of Antibodies to SARS-CoV-2 in 10 Sites in the United States, March 23-May 12, 2020.* JAMA Intern Med. *Published online July 21, 2020. doi:10.1001/jamainternmed.2020.4130/*

- e = 0.4 /margin of errors/

This would be done in an attempt to include participants **over a range of ages** in order to determine and compare age-specific sero-prevalence. This will ensure that the following 10 age groups are reported: 1-4, 5-9, 10-14, 15-19, 20-29, 30-39, 40-49, 50-59, 60-69, 70+. Sampling within each stratum will be done by systematic sampling.

### Table 1. Age group fraction of the study population

| Age, years | Number of Total Population | Fraction | Sample size by age groups |
| --- | --- | --- | --- |
| 0-4 yrs | 391,125. | 12 | 600 |
| 5-9 yrs | 358,379. | 11 | 550 |
| 10-14 yrs | 252,548. | 8 | 400 |
| 15-19 yrs | 228,402. | 7 | 350 |
| 20-29 yrs | 539,521. | 17 | 850 |
| 30-39 yrs | 539,323. | 17 | 850 |
| 40-49 yrs | 413,694. | 13 | 650 |
| 0-59 yrs | 299,756. | 9 | 450 |
| 60-69 yrs | 138,097. | 4 | 200 |
| 70 +yrs | 77,634. | 2 | 100 |
| Total | 3,238,479. |  | 5000 |

We propose to take a population of 600 or 900 depending on the population size in the region and 2300 in the capital city where 60% of the Mongolian population resides, totaling 5000 samples.

### Table 2. Number of Clusters selected from each provinces and Ulaanbaatar city and sample size of the study

| **Region** | **Selected provinces/ and Districts** | **Number of clusters** | | | **Sample size** |
| --- | --- | --- | --- | --- | --- |
|  |  | **In a province center** | **In a soum** | **Total** |  |
| Eastern region | Dornod aimag | 1 | 2 | 3 | 300 |
|  | Khentii aimag | 1 | 2 | 3 | 300 |
| **Sub total** | | | | **6** | **600** |
| Central region | Selenge aimag | 1 | 2 | 3 | 300 |
|  | Umnugobi aimag | 1 | 2 | 3 | 300 |
|  | Dornogobi aimag | 1 | 2 | 3 | 300 |
| **Sub total** | | | | **9** | **900** |
| Khangai region | Erdenet city | 1 | 2 | 3 | 300 |
|  | Bayankhongor aimag | 1 | 2 | 3 | 300 |
| **Sub total** | | | | **6** | **600** |
| Western region | Bayan-Ulgii aimag | 1 | 2 | 3 | 300 |
|  | Zavkhan aimag | 1 | 2 | 3 | 300 |
| **Sub t otal** | | | | **6** | **600** |
| Capital city, districts | Songinokhairkhan district | 5 | | 5 | 500 |
|  | Khan-Uul district | 3 | | 3 | 300 |
|  | Bayangol district | 4 | | 4 | 400 |
|  | Bayanzurkh district | 4 | | 4 | 400 |
|  | Chingeltei district | 4 | | 4 | 400 |
|  | Sukhbaatar district | 3 | | 3 | 300 |
|  | |  |  | **23** | **2300** |
| **TOTAL** | | | | **50** | **5000** |

Study population

*Selection of provinces and cities:*

Mongolia is geographically divided into 5 regions: West, Khangai, Central, East, and Ulaanbaatar based on the residential area of population.

All 21 provinces (aimags) of Mongolia are listed below by region:

- Western region provinces include: Bayan-Ulgi (1), Gobi Altai (2), Zavkhan (3), Khovd (4), Uvs (5)
- Khangai region provinces include: Arkhangai (1), Bayankhongor (2), Bulgan (3), Uvurkhangai (4), Khuvsgul (5)
- Central region provinces include: Umnugobi (1), Dornogobi (2), Dundgobi (3), Gobisumber (4), Tuv (5), Selenge (6), Darkhan-Uul (7), Orkhon (8)
- Eastern region provinces include: Khentii (1), Dornod (2), Sukhbaatar (3)

From above 21 provinces Two provinces from each region and Ulaanbaatar city were selected randomly by using the “randbetween” tool in MS Excel.

The study population will be citizens and randomly selected from the following provinces

(8) and cities (2):


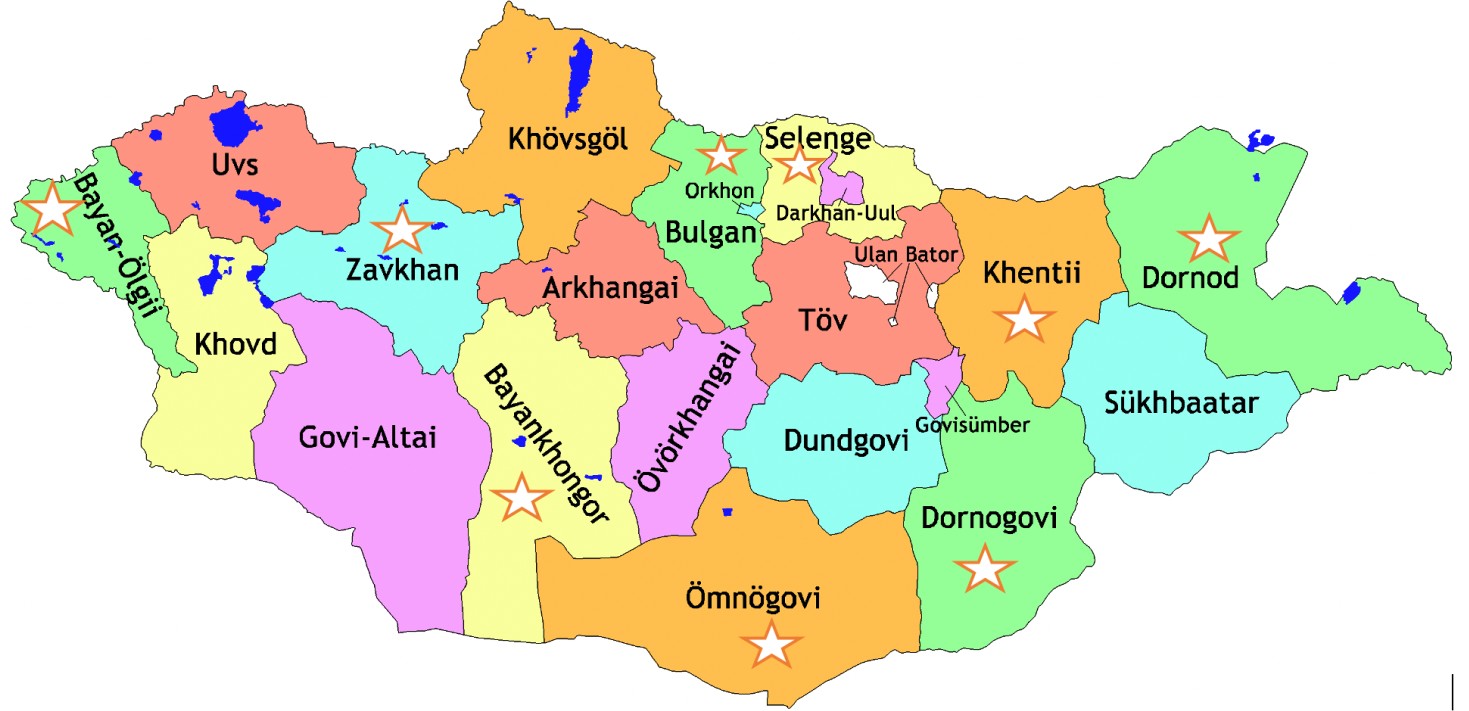
Dornod, Khentii, Umnugobi, Dornogovi, Selenge, Bayankhongor, Bayan-Ulgii and Zavkhan aimags and Erdenet, Ulaanbaatar cities (selected provinces marked with star in Figure 2). These 9 areas cover almost half of the 21 provinces of Mongolia and represent both high and low incidence areas along with urban and rural populations of Mongolia. Additionally, except Ulaanbaatar city, Mongolia has only two minor cities: Darkhan and Erdenet. From these two cities, Erdenet was also selected randomly.

### Fig 4. Selected provinces for the survey

Ulaanbaatar ‘s team (working group) will be the central coordinating site for all other

nine study sites which will provide overall technical support, guidance and will coordinate together with a regional team.

**Table 3. Soum and khoroo level selection**

| **Sites** | **Provinces** | **Selected soums and khoroos** |
| --- | --- | --- |
| Eastern site | Dornod aimag | Province center: Kherlen 1^st^ khoroo,  Soums: Tsagaan Ovoo, Bayandun |
|  | Khentii aimag | Province center: 2^nd^ khoroo,  Soums: Batnorov, Bayankhutag |
| **Total=600** | | |
| Central site | Selenge aimag | Province center: 1^st^ and 3^rd^ khoroo  Soums: Altanbulag, Khushaat |
|  | Umnugobi aimag | Province center: 2^nd^ khoroo  Soums: Khankhongor, Khurmen |
|  | Dornogobi aimag | Province center: 1^st^ khoroo  Soums: Urgun, Zamiin uud |
| **Total=900** | | |
| Khangai sites | Erdenet city | Province center: 1^st^ and 3^rd^ khoroo  Soums: Jargalant |
|  | Bayankhongor aimag | Province center: 1^st^ khoroo,  Soums: Bogd, Jinst |
| **Total=600** | | |
| Western sites | Bayan –Ulgii | Province center: 1^st^ khoroo  Soums: Sagsai, Ulaankhus |
|  | Zavkhan aimag | Province center: 2^nd^ khoroo  Soums – Tsagaankhairkhan , Аldarkhaan |
| **Total=600** | | |
| Capital city | Songinokhairkhan district | Khoroo-9, 19, 17, 1, 12 |
|  | Khan-Uul district | Khoroo-1, 5, 13 |
|  | Bayangol district | Khoroo-3, 13, 16, 20 |
|  | Bayanzurkh district | Khoroo-2, 13, 20, 24 |
|  | Chingeltei district | Khoroo-1, 6, 14, 19 |
|  | Sukhbaatar district | Khoroo-1,11,17 |
| **Total=2300** | | |
| **Grand total=5000** | | |

**Epidemiological indicators**

The table below provides an overview of the epidemiological parameters that can be measured as part of this investigation.

Table 4: Indicators to inform investigation objectives

| **Objective** | **Parameter** | **Definition** | **Data source to calculate the parameters**  **concerned** | **Comments, limitations** |
| --- | --- | --- | --- | --- |
| **1. Measure the sero- prevalence of antibodies to COVID-19 in the general population by age group** in order to ascertain the cumulative population immunity | Sero-prevalenc e (population and  age-specific) | The proportion of individuals per age strata who show sero-positivity for COVID-19 virus infection | - Seropositivity - Age group | - Population seroprevalence will be calculated using direct standardization methods, so that the proportion is adjusted for any difference in the age stratification of the participants and the overall population  **-** Age-specific seroprevalence is same as ***age-specific attack rate*** and ***cumulative incidence*.**  - If data is collected, seroprevalence by different groups (e.g. geography, profession, residence) will be an important sub-analysis  depending on numbers we get. |

| **2. Estimate the fraction of asymptomatic or pre- symptomatic/ subclinical infections in the population and by age group.** | Asymptomatic Fraction (proportion of cases that are asymptomatic) | The proportion of individuals who reported no symptoms of COVID- 19 infection of individuals seropositive for COVID- 19 | - Sero- positivity - Reported symptom s | - The numerator is the number of individuals reporting no symptoms and the denominator is the total number of individual’s sero-positive for COVID-19. |
| --- | --- | --- | --- | --- |
|  | Fraction severe disease | The number individuals with severe infection | - Sero-positivity - Reported symptoms - Age group | - Severe disease will be defined (like hospitalization). - The number individuals with severe infection divided by the number with COVID-19 infection as determined by sero-positivity. |
| **3. Determine risk factors for infection** by comparing the exposures of infected and non-infected individuals | Population groups most at risk | The identification of groups who are most vulnerable to COVID-19 virus infection (e.g. age groups, gender, occupation) | - Sero-positivity - Reported symptom s - Exposure of interest (e.g. age group) | - May only be an early signal, a nested case-control study could be conducted to evaluate risk factors for infection if required. |
| **4. Contribute to determine the case fatality ratio** | Case fatality ratio | The proportion of individuals with fatal outcome for COVID-19 Infection | - Sero-positivity - Mortality Age group | - We will use longitudinal cohort investigations to record sufficient events (i.e. deaths) - May require extended follow- up to determine outcome of those with COVID-19 infection beyond 2 years if required at that time for long term sequelae |
| **5. To contribute to an improved understanding of antibody kinetics following COVID-19 infection.** | Serological response to infection | The change in serum level of specific antibodies to COVID-19 virus (*Increase in titer*) | - Antibody titer | - Changes in titers would be calculated using geometric mean titers (GMTs) and PRNT |


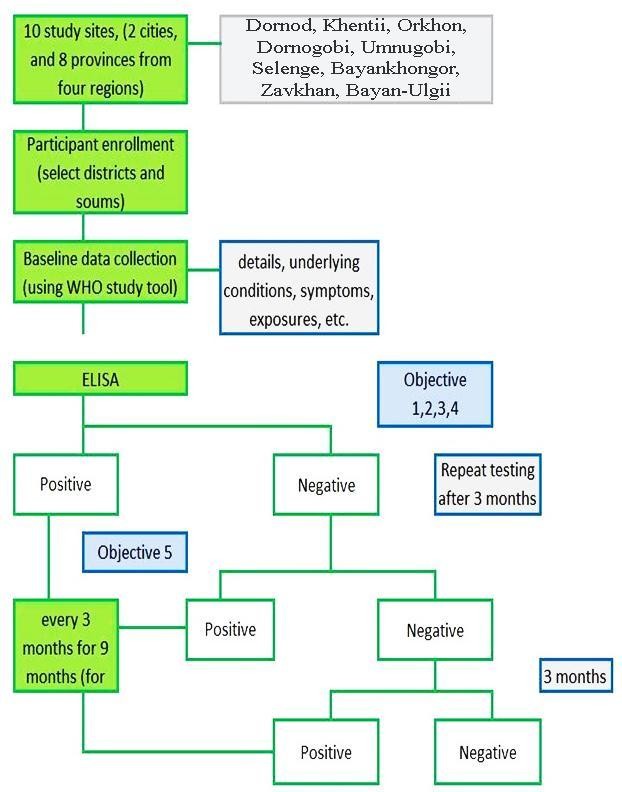
We will follow all positives every three months for nine months to see the antibody titers for antibody kinetics. **(Fig. 3)**

## Fig 3: Case investigation algorithm and summary of data-collection tool

Samples will be drawn and would be transported, stored and tested as per standard protocol of the test kit used. Positive samples will be collected every three months for at least 9 months to see persistence of antibody titer. We will be doing the ELISA test

in Ulaanbaatar at University hospital laboratory, MNUMS.

1 Riley S, Kwok KO, Wu KM et al. Epidemiological characteristics of 2009 (H1N1) pandemic influenza based on paired sera from a longitudinal community cohort study. *PLoS Med*. 2011 Jun;8(6):e1000442

### Eligibility criteria Inclusion criteria:

All individuals identified for recruitment into the investigation, irrespective of age,

irrespective of acute or prior COVID-19 infection.

### Exclusion criteria:

Refusal to give informed consent, or contraindication to venipuncture The confirmed cases only imported by international charter flight

### Data and specimen collection and transport

Information about the subject's age, gender, area of residence, occupation, workplace; number of persons in the household, travel, exposure details, and (symptoms of respiratory illness if any, duration of disease, medical consultation, treatment, hospitalization and outcome) as per WHO protocol will be collected using a paper based/ tablet-based study tool. We would be contributing this data to global data from UNITY studies.

Data and specimen collection will be repeated in selected individuals who were participants with negative results at baseline will be followed up after 3 months, and if further negative, followed up again at another 3 months (total number of samples taken will be 3, at months 0, 3 and 6). If the participant seroconverts at any of the time periods, they will be followed up for one year (total number of samples depends on when seroconversion takes place, at minimum if the result is positive at baseline, then follow- up will be months 3, 6 and 9 for a total of 4 samples taken), and then in negatives at 6 months. Longitudinal follow-up of positive cases will be conducted to understand immunity kinetics.

Data and specimen collection at each site will be conducted in primary health care settings (family and soum health care center). To cover the unreached population in remote areas data and specimen collection will be conducted in households.

Each participant recruited and sampled for the investigation would be asked to complete a questionnaire which covers demographic, clinical and exposure information. An example of an investigation questionnaire which will be used can be found in the Appendix: *Form 1 “Reporting form for each participant”*.

The data collection also includes the reported COVID-19 laboratory testing by investigation subjects.

A serum sample will be collected from each participant upon recruitment into the investigation. Totally, 5 ml blood from adults and 3 ml blood from children will be collected each time and will make 3 aliquots for lab testing. All those involved in the collection and transportation of specimens will be trained in safe handling practices and spill decontamination procedures. For details regarding the transport of samples collected and infection control advice, we will follow case management algorithm and laboratory guidance in the country or WHO laboratory guidance, available on the [WHO website.](https://www.who.int/emergencies/diseases/novel-coronavirus-2019/technical-guidance/laboratory-guidance)

For each biological sample collected, the time of collection, the conditions for transportation and the time of arrival at the study laboratory will be recorded. Specimens will be transported in a portable refrigerator for sample transportation and reach the laboratory as soon as possible after collection. In case of need to store specimens in provincial and soum level, medical freezers of soum and family health centers will be used. All soums selected to the study have capacity of centralized electricity. In case of electricity off, all soums have portable generators.

Serum will be separated from whole blood and transported at 4°C or frozen to -20°C or lower (at -80°C) and will be shipped on dry ice if required.

### Laboratory procedures

Laboratory procedures involving sample manipulation will be carried out in a bio-safety cabinet (BSC) in the University hospital laboratory.

Serum samples will be screened for the presence of COVID-19 virus specific antibodies using serological testing. Tests for total antibodies for SARS-CoV-2 will be tested by Wantai Total Ab ELISA kit and Florian Krammer’s ELISA from Kantaro (New York) ELISA kits. The Kantaro kit’s a 2 step ELISA, 1^st^ on RBD antigen and then the positives are tested against the spike antigen which is WHO procured.

ELISA testing would be carried out in a facility with **at bio-safety level 2 (BSL-2)** capacity. Serum samples will be stored at -80°C. Neutralizing antibody testing will not be applied as there are no facilities available in Mongolia. Stored specimens may be tested in the future should tests become available for detecting neutralizing antibodies by ELISA. We would **aliquot** samples prior to freezing, to minimize freeze thaw cycles.

The storage of serum specimens in domestic frost-free freezers should be avoided, owing to their wide temperature fluctuations.

### Ethical considerations

Ethical clearance will be obtained from the Bio-Ethics and Research Committee, MOH before the start of study.

*Informed consent*

The purpose of the investigation will be explained to all individuals identified for recruitment into the investigation. Written informed consent will be obtained from all individuals willing to participate in the investigation before any procedure is performed as part of the investigation by a trained member of the investigation team. Consent for children under the legal age of consent will be obtained from a parent or legal guardian. Each participant must be informed that participation in the investigation is voluntary and that s/he is free to withdraw, without justification, from the investigation at any time without consequences and without affecting professional responsibilities.

Informed consent will seek approval to collect blood and epidemiological data for the intended purpose of this investigation, that samples may be shipped outside of the country for additional testing and that samples may be used for future research purposes. *Risks and benefits for subjects*

This investigation poses minimal risk to participants, involving the collection of small amounts (3 ml in adults, 2 ml in children) of blood. The primary benefit of the study is

indirect in that data collected will help improve and guide efforts to understand extent of COVID-19 virus infection and may prevent further transmission of the virus.

*Confidentiality*

Participant confidentiality will be maintained throughout the investigation. All subjects who participate in the investigation will be assigned a study identification number by the investigation team for the labeling of questionnaires and specimens. The link of this identification number to individuals will be maintained by the investigation team and the Ministry of Health (or equivalent) and will not be disclosed elsewhere.

If the data is shared by the implementing organization to WHO or any agency or institution providing support for data analysis, data shared will include only the study identification number and not any personal identifiable information.

*Prevention of COVID-19 infection among study personnel*

All personnel involved in the investigation would be trained in infection prevention and control procedures (standard contact and droplet precautions, as determined by national guidelines). These procedures should include proper hand hygiene and the correct use of surgical masks, if necessary, not only to minimize their own risk of infection when in close contact with individuals with COVID-19 infection, but also to minimize the risk of spread among other participants in the investigation.

### Timeline

| **Activities** | **0-1**  **mo** | **1mo** | **3-4**  **mo** | **6-7**  **mo** | **9-10**  **mo** | **11-**  **12**  **mo** |
| --- | --- | --- | --- | --- | --- | --- |
| - Protocol review meeting and ethical clearance - Team   recruitment, sensitization and trainings |  |  |  |  |  |  |
| - Field visits, participant enrolment - Data collection, blood sampling - Database creation, data entry |  |  |  |  |  |  |
| - 2^nd^ round of data collection (for Incidence) - Field visits, participant enrolment - Data collection,   blood sampling, data entry   - Follow-up of positive cases for objective 4 and 5 |  |  |  |  |  |  |
| **Midterm analysis** | | | | | | |
| - 3^rd^ round of data (for Incidence) - Field d visits, participant enrolment - Data collection,   blood sampling, data entry   - Follow-up of positive cases   for objective 4 and 5 |  |  |  |  |  |  |
| - 4^th^ round of data (for Incidence) - Field d visits, participant enrolment - Data collection,   blood sampling, data entry   - Follow-up of positive cases   for objective 4 and 5 |  |  |  |  |  |  |
| **INTERIM ANALYSIS** | | | | | | |
| - All reports and final data handover |  |  |  |  |  |  |

1. **References**

WHO Situation reports

[https://www.who.int/emergencies/diseases/novel-coronavirus-2019/situation-](https://www.who.int/emergencies/diseases/novel-coronavirus-2019/situation-reports/) [reports/](https://www.who.int/emergencies/diseases/novel-coronavirus-2019/situation-reports/)

The Unity studies: early investigations protocols [https://www.who.int/emergencies/diseases/novel-coronavirus-2019/technical-](https://www.who.int/emergencies/diseases/novel-coronavirus-2019/technical-guidance/early-investigations) [guidance/early- investigations](https://www.who.int/emergencies/diseases/novel-coronavirus-2019/technical-guidance/early-investigations)

Surveillance, rapid response team and case definitions [https://www.who.int/emergencies/diseases/novel-coronavirus-2019/technical-](https://www.who.int/emergencies/diseases/novel-coronavirus-2019/technical-guidance/surveillance-and-case-definitions) [guidance/surveillance-and-case-definitions](https://www.who.int/emergencies/diseases/novel-coronavirus-2019/technical-guidance/surveillance-and-case-definitions)

Laboratory

[https://www.who.int/emergencies/diseases/novel-coronavirus-2019/technical-](https://www.who.int/emergencies/diseases/novel-coronavirus-2019/technical-guidance/laboratory-guidance) [guidance/laboratory-guidance](https://www.who.int/emergencies/diseases/novel-coronavirus-2019/technical-guidance/laboratory-guidance)

Clinical care

[https://www.who.int/emergencies/diseases/novel-coronavirus-2019/technical-](https://www.who.int/emergencies/diseases/novel-coronavirus-2019/technical-guidance/patient-management) [guidance/patient- management](https://www.who.int/emergencies/diseases/novel-coronavirus-2019/technical-guidance/patient-management)

Infection prevention and control / WASH [https://www.who.int/emergencies/diseases/novel-coronavirus-2019/technical-](https://www.who.int/emergencies/diseases/novel-coronavirus-2019/technical-guidance/infection-prevention-and-control) [guidance/infection- prevention-and-control](https://www.who.int/emergencies/diseases/novel-coronavirus-2019/technical-guidance/infection-prevention-and-control)

Risk communications and community engagement [https://www.who.int/emergencies/diseases/novel-coronavirus-2019/technical-](https://www.who.int/emergencies/diseases/novel-coronavirus-2019/technical-guidance/risk-communication-and-community-engagement) [guidance/risk- communication-and-community-engagement](https://www.who.int/emergencies/diseases/novel-coronavirus-2019/technical-guidance/risk-communication-and-community-engagement)

Examples of sero-epidemiological studies <https://www.medrxiv.org/content/10.1101/2020.04.19.20071563v1> <https://www.medrxiv.org/content/10.1101/2020.04.13.20060467v1> <https://www.medrxiv.org/content/10.1101/2020.04.14.20062463v1>

WHO Scientific brief "Immunity passports" in the context of COVID-19 [https://www.who.int/publications-detail/immunity-passports-in-the-context-of-covid-](https://www.who.int/publications-detail/immunity-passports-in-the-context-of-covid-19) [19](https://www.who.int/publications-detail/immunity-passports-in-the-context-of-covid-19)

# Appendix: Questionnaires

### Population-based age-stratified sero-epidemiological investigation protocol for COVID-19 infection

**Form 1: Reporting form for each participant**


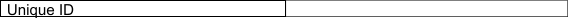


□ Alive □ Dead □ Unknown/lost to follow-up

**Current status**

| **1. Data Collector Information** | |
| --- | --- |
| Name of data collector |  |
| Data collector Institution |  |
| Data collector telephone number |  |
| Mobile number |  |
| Email |  |
| Form completion date(dd/mm/yyyy) | _/ / _ |
| Date of interview with informant  (dd/mm/yyyy) | _/ / _ |

| **2. Identifier information** | |
| --- | --- |
| First name |  |
| Family name |  |
| Sex | □ Male □ Female □ Not known |
| Date of birth (dd/mm/yyyy) | / /  □ Unknown |
| Age (years, months) |  |
| Telephone (mobile) number |  |
| Email |  |
| Country of residence |  |
| Nationality |  |
| Ethnicity (optional) |  |
| Occupation | □ HCW (including nursery and quarantine facility)  if yes, please write in detail |
|  | And please complete Form 3  □Non HCW  if yes, please write in detail |
|  |  |

| Have you had contact with a anyone with suspected or confirmed COVID-19 virus infection? | □ Yes □ No □ Unknown  If Yes, dates of last contact (DD/MM/YYYY):  _/ / _ |
| --- | --- |

| **3. Symptom history** | |
| --- | --- |
| In the past (…) months, have you had any of the following: | |
| Fever (≥38 °C) or history of fever | □ Yes □ No □ Unknown |
| Sore throat | □ Yes □ No □ Unknown |
| Runny nose (rhinorrea) | □ Yes □ No □ Unknown |
| Cough | □ Yes □ No □ Unknown |
| Shortness of breath (dyspnea) | □ Yes □ No □ Unknown |
| Other respiratory symptoms | □ Yes □ No □ Unknown If Yes, specify: |
| Chills | □ Yes □ No □ Unknown |
| Vomiting | □ Yes □ No □ Unknown |
| Nausea | □ Yes □ No □ Unknown |
| Diarrhea | □ Yes □ No □ Unknown |
| Headache | □ Yes □ No □ Unknown |
| Rash | □ Yes □ No □ Unknown |
| Conjunctivitis | □ Yes □ No □ Unknown |
| Muscle aches | □ Yes □ No □ Unknown |
| Joint ache(myalgia) | □ Yes □ No □ Unknown |
| Loss of appetite | □ Yes □ No □ Unknown |
| Loss of smell (anosmia) | □ Yes □ No □ Unknown |
| Loss of taste (ageusia) | □ Yes □ No □ Unknown |
| Nose bleed | □ Yes □ No □ Unknown |
| Fatigue | □ Yes □ No □ Unknown |
| Seizures | □ Yes □ No □ Unknown |
| Altered consciousness | □ Yes □ No □ Unknown |
| Other neurological signs | □ Yes □ No □ Unknown If Yes, specify: |
| Other symptoms | □ Yes □ No □ Unknown If Yes, specify: |

| **4. Patient symptoms: complications** | |
| --- | --- |
| Did any of these symptoms require you to seek medical attention? | □ □ No □ Unknown  Yes |
| Did any of these symptoms require you to miss work or  school? | □ □ No □ Unknown Yes |
| Hospitalization: Did any of these  symptoms require you to be hospitalized? | □ Yes □ No □ Unknown |

**Form 2: Assessment of potential risk factors for coronavirus disease 2019 (COVID-19) among health workers in a health care setting**

| **1. Adherence to infection prevention and control (IPC) measures information** | | |
| --- | --- | --- |
| Smoker | □ Yes □ No |  |
| Occupation in health care facility | - Medical doctor - Registered nurse (or equivalent) - Assistant nurse, nurse technician (or equivalent) - Radiology/x-ray technician - Phlebotomist - Physical therapist - Nutritionist/dietitian   **Other health personnel**:   - Laboratory personnel - Admission/reception clerks - Patient transporters - Catering staff - Cleaners |  |

| **2. Adherence to infection prevention and control (IPC) measures information** | |
| --- | --- |
| What was the date of your most recent IPC training within the health care facility (dd/mm/yyyy) | / / |
| How much cumulative IPC training (standard precautions, additional precautions) have you had at this health care facility | - Less than 2 hours - More than 2 hours |
| Do you follow recommended hand hygiene practices? | - Always, as recommended - Most of the time - Occasionally - Rarely |
| Do you use alcohol-based hand rub or soap and water before touching a patient? | - Always, as recommended - Most of the time - Occasionally - Rarely |
| Do you use alcohol-based hand rub or soap and water before cleaning/aseptic procedures? | - Always, as recommended - Most of the time - Occasionally - Rarely |
| Do you use alcohol-based hand rub or soap and water after (risk of) body fluid exposure? | - Always, as recommended - Most of the time - Occasionally - Rarely |
| Do you use alcohol-based hand rub or soap and water after touching a patient? | - Always, as recommended - Most of the time - Occasionally - Rarely |
| Do you use alcohol-based hand rub or soap and water after touching a patient’s surroundings? | - Always, as recommended - Most of the time - Occasionally - Rarely |
| Do you follow IPC standard precautions when in contact with any patient? | - Always, as recommended - Most of the time - Occasionally - Rarely - I don’t know what IPC standard precautions are |
| Do you wear PPE when indicated?  (PPE includes: medical mask, face shield, gloves, goggles/glasses, gown, coverall, head cover, respirator (for example, N95 or equivalent) and shoe covers) | - Always, according to the risk assessment - Most of the time, according to the risk assessment - Occasionally - Rarely |
| Is PPE available in sufficient quantity in the health  care facility? | □ Yes □ No □ Unknown |
